# Depletion of Arg/Abl2 improves endothelial cell adhesion and prevents vascular leak during inflammation

**DOI:** 10.1007/s10456-021-09781-x

**Published:** 2021-03-26

**Authors:** Joana Amado-Azevedo, Anne-Marieke D. van Stalborch, Erik T. Valent, Kalim Nawaz, Jan van Bezu, Etto C. Eringa, Femke P. M. Hoevenaars, Iris M. De Cuyper, Peter L. Hordijk, Victor W. M. van Hinsbergh, Geerten P. van Nieuw Amerongen, Jurjan Aman, Coert Margadant

**Affiliations:** 1 Department of Physiology, Amsterdam Cardiovascular Sciences, Amsterdam University Medical Center, Amsterdam, The Netherlands; 2 Sanquin Research, Amsterdam, The Netherlands; 3 Department of Pulmonology, Amsterdam Cardiovascular Sciences, Amsterdam University Medical Center, Amsterdam, The Netherlands; 4 Angiogenesis Laboratory, Department of Medical Oncology, Cancer Center Amsterdam, Amsterdam University Medical Center, Amsterdam, The Netherlands

**Keywords:** Arg/Abl2, Endothelial barrier function, Inflammation, Integrins, Vascular leak, VE-cadherin

## Abstract

Endothelial barrier disruption and vascular leak importantly contribute to organ dysfunction and mortality during inflammatory conditions like sepsis and acute respiratory distress syndrome. We identified the kinase Arg/Abl2 as a mediator of endothelial barrier disruption, but the role of Arg in endothelial monolayer regulation and its relevance in vivo remain poorly understood. Here we show that depletion of Arg in endothelial cells results in the activation of both RhoA and Rac1, increased cell spreading and elongation, redistribution of integrin-dependent cell-matrix adhesions to the cell periphery, and improved adhesion to the extracellular matrix. We further show that Arg is activated in the endothelium during inflammation, both in murine lungs exposed to barrier-disruptive agents, and in pulmonary microvessels of septic patients. Importantly, Arg-depleted endothelial cells were less sensitive to barrier-disruptive agents. Despite the formation of F-actin stress fibers and myosin light chain phosphorylation, Arg depletion diminished adherens junction disruption and intercellular gap formation, by reducing the disassembly of cell-matrix adhesions and cell retraction. In vivo, genetic deletion of Arg diminished vascular leak in the skin and lungs, in the presence of a normal immune response. Together, our data indicate that Arg is a central and non-redundant regulator of endothelial barrier integrity, which contributes to cell retraction and gap formation by increasing the dynamics of adherens junctions and cell-matrix adhesions in a Rho GTPase-dependent fashion. Therapeutic inhibition of Arg may provide a suitable strategy for the treatment of a variety of clinical conditions characterized by vascular leak.

**Supplementary Information:**

The online version contains supplementary material available at 10.1007/s10456-021-09781-x.

## Introduction

Endothelial barrier disruption and vascular leak are hallmarks of a variety of life-threatening conditions including sepsis, acute respiratory distress syndrome (ARDS), and COVID-19, and importantly contribute to the morbidity and mortality of critically ill patients [1–3]. Despite the serious adverse clinical outcome associated with vascular leak, no treatment is currently available to reverse endothelial barrier disruption [2, 4].

Barrier function critically depends on intercellular adhesion complexes including adherens junctions (AJs), formed by vascular endothelial (VE)-cadherin. In addition, barrier function requires cell adhesion to the basement membrane maintained by integrins, a family of α β heterodimeric transmembrane receptors crucial for the assembly of cytoskeleton-associated adhesion complexes such as focal adhesions (FAs) [4]. During injury or inflammation, endothelial barrier disruption is induced by a variety of agents including lipopolysaccharide (LPS), inflammatory cytokines, or thrombin, which trigger a rapid and transient increase in permeability by activation of the small GTPase RhoA, leading to a Rho kinase-dependent increase in myosin light chain (MLC) phosphorylation and the formation of actin stress fibers [5–9]. The resulting cytoskeletal contractility transforms stable AJs into remodeling junctions, which is associated with VE-cadherin internalization, thus promoting cell retraction and increasing endothelial permeability [7–10]. This process is counteracted by cell spreading dependent on the GTPase Rac1, which promotes the assembly of peripheral cell-matrix adhesions and AJs [7, 11–13]. Thus, a tight balance between Rac1 and RhoA activities regulates cell spreading versus contractility, and is essential for normal barrier regulation.

Extensive crosstalk exists between integrins, Rho GTPases, and cell-cell adhesions. Vascular endothelial cells express several integrins of the β1 subfamily that bind to ligands in the extracellular matrix, which reinforces VE-cadherin-dependent cell-cell adhesion and contributes to barrier function in quiescent endothelium [14–16]. Furthermore, regulators of integrins such as talin, which binds integrin β-cytoplasmic tails to stimulate integrin activation, also reinforce AJs and are essential for endothelial barrier function [17]. However, in recent years it has become clear that β1 integrins, in particular α5β1, can also promote barrier disruption in pathological conditions such as inflammation [18, 19]. Integrin α5β1 binds to fibronectin (FN), which is strongly upregulated during injury, remodeling, and inflammation [20, 21]. The interaction between FN and α5β1 induces robust RhoA activation, which in turn triggers the assembly of FN fibrils and associated fibrillar adhesions (FBs), a special kind of tension-bearing adhesion complexes located in the cell center that facilitate cytoskeletal contractility and cell contraction [22–24]. Indeed, we and others have previously found that α5β1 disrupts cadherin-dependent cell-cell junctions through RhoA-dependent contractility, in a variety of cell types including endothelial cells [18, 19, 25, 26]. Furthermore, blocking β1 integrins was recently shown to protect from LPS-induced barrier disruption and vascular leakage in mice [18, 19]. Thus, integrins exert dual functions on endothelial barrier function, through their effects on Rho GTPases and crosstalk with cell-cell junctions.

We have previously found in pre-clinical studies that the tyrosine kinase inhibitor imatinib (Gleevec®) preserves endothelial barrier integrity by inhibition of the tyrosine kinase Abl-related gene (Arg/Abl2) [27], corroborating clinical reports which demonstrated that imatinib reverses vascular leak and pulmonary edema in patients [28–31]. These data suggest that Abelson-family kinases are potential targets for the treatment of vascular leak [30], and we have recently completed clinical trials with imatinib to prevent pulmonary vascular leak in patients with respiratory failure due to COVID-19 (EudraCT # 2020-001236-10).

Imatinib treatment in endothelial and other cells induced an increase in FA numbers in vitro, in particular at the cell periphery, suggesting that the Abelson kinases regulate the organization of cell-matrix adhesions [27, 32]. Furthermore, knockdown of both kinases in epithelial cells revealed that they regulate the stability of AJs, through their effects on Rac and Rho activities [33]. The combined targeted deletion of the genes encoding c-Abl (Abl1) and Arg in mice is embryonically lethal by E9.5, due to disrupted vascular development and endothelial cell apoptosis [34]. Finally, the endothelial-specific deletion of the gene encoding c-Abl in heterozygous Arg^+/−^ mice protects against endothelial barrier disruption [35], in line with other studies showing that c-Abl regulates barrier function [36–38]. However, the specific role of Arg in endothelial barrier regulation is poorly understood and remains unexplored. Intriguingly, studies in fibroblasts have shown that Arg phosphorylates p190RhoGAP, which inhibits RhoA activation and RhoA-dependent FA turnover [39, 40]. Other Arg substrates associated with integrin-dependent cell-matrix adhesions include cortactin, Crk and CrkL, as well as the cytoplasmic tail of the integrin β1 subunit [41–43].

In this study, we find that Arg is a key regulator of Rho GTPase activation, cell-matrix adhesion, and cell-cell adhesion in endothelial cells. Arg promotes RhoA-dependent cell retraction, VE-cadherin internalization, and endothelial barrier disruption. Depletion of Arg protects against barrier disruption in vitro, and Arg-deficient mice are protected from LPS-induced vascular leak and pulmonary edema. Thus, our data suggest that Arg inhibition improves endothelial barrier function, and constitutes a strategy for the treatment of respiratory failure caused by endothelial dysfunction.

## Results

### Arg regulates Rac1 activation, cell spreading, and organization of cell-matrix adhesions in endothelial cells

To investigate the role of Arg in endothelial cells, we depleted Arg from human umbilical vein endothelial cells (HUVECs) by RNA interference. Arg expression was reduced up to 85% at the mRNA and protein level, as compared to control cells that received scrambled sequences, while c-Abl expression was not significantly downregulated (Suppl. Fig. S1a, b).

**Fig. 1:**
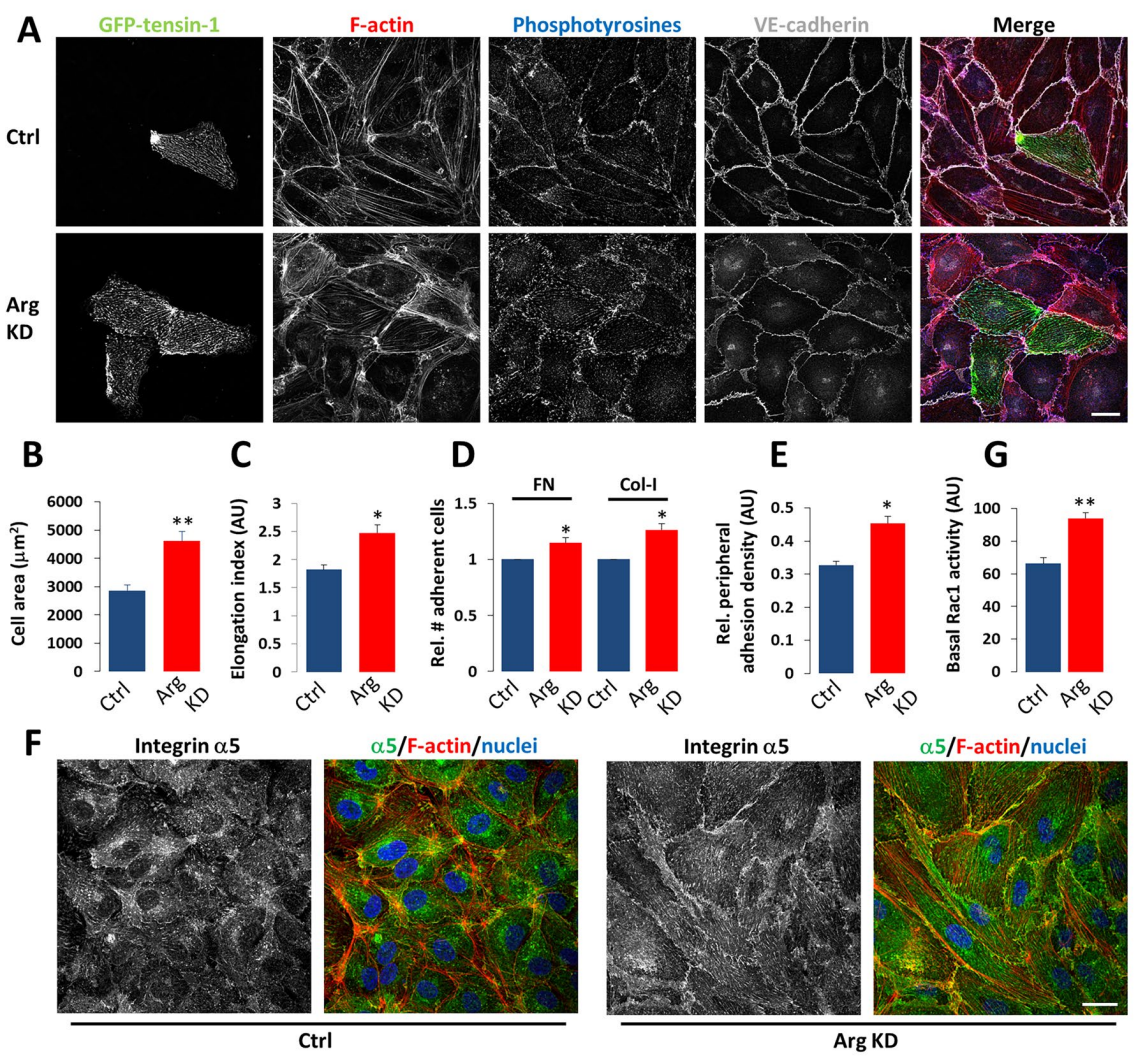
Arg controls integrin-dependent cell-matrix adhesions, cell adhesion and spreading, and Rho GTPase activation in HUVECs. **a** Representative confocal images showing the distribution of GFP-tensin-1 (*green*), F-actin (*red*), phosphotyrosines (*blue*), and VE-cadherin (*grey*) in control and Arg-depleted HUVEC monolayers. Bar, 30 μm. **b** Quantification of HUVEC cell spreading. **c** Quantification of length/width ratio. **d** HUVECs were seeded in serum-free medium on Col-I (3 μg/ml) or FN (5 μg/ml). After 10 mins, non-adherent cells were washed away and the adherent fraction was quantified. Means + SEM of *n* = 4 experiments. **e** Quantification of peripheral distribution of integrin α5 quantified from confocal images as in (**f**), as described in Methods. Means + SEM of *n* = 20 cells/condition. Rel., relative. **f** Representative confocal images of integrin α5 (*green*) and F-actin (*red*) in control and Arg-depleted HUVEC monolayers. Nuclei are stained with DAPI (*blue*). Bar, 40 µm. **g** Basal Rac1 activity as assessed in pulldown assays. Data are means + SEM from *n* = 3. *P < 0.05; **P< 0.01. AU, arbitrary units.

Arg depletion induced a number of alterations in HUVEC morphology and the organization and distribution of cell-matrix adhesions. Arg-depleted cells became larger and on average more elongated than control cells (Fig. 1a–c). This phenomenon was observed in monolayers but even more prominently in sparsely seeded cells, thus excluding indirect effects resulting from potential differences in cell number or confluency, and suggesting that integrin- and/or Rac-dependent cell spreading were enhanced (Suppl. Fig. S2a). In line with this, downregulation of Arg induced increased HUVEC adhesion to the β1 integrin ligands Col-1 and FN in adhesion assays (Fig. 1d). Overexpression of Arg has been shown to promote phosphorylation of Y783 in the β1 cytoplasmic tail, which is important for talin binding and integrin activation [42]. Therefore, we next assessed the activation status of β1, using the antibody 9EG7 that recognizes the high-affinity conformation of β1 integrins. The proportion of active β1 integrins was unchanged in Arg-depleted HUVECs, but the total amount of β1 integrins was slightly increased, in line with the observation that expression of an active Arg mutant reduced β1 expression in epithelial cells [44] (Suppl. Fig. S2b, c). Next we examined whether Arg phosphorylates Y783 in the β1 cytoplasmic tail in HUVECs, and whether this was affected by thrombin, which promotes RhoA-dependent endothelial barrier disruption [8]. Y783 phosphorylation was only detectable when cells were treated with Na3VO4 to inhibit phosphatases, and a transient decrease followed by recovery was observed upon thrombin addition (Suppl. Fig. S2d). However, the depletion of Arg had no apparent effect (Suppl. Fig. S2d).

We then assessed whether Arg affects the organization or distribution of cell-matrix adhesions, either by ectopic expression of GFP-tensin-1, a marker for FBs, or using antibody 4G10 that detects phosphorylated tyrosines, a marker for FAs (Fig. 1a). FA formation seemed enhanced in Arg-depleted HUVECs, especially at the cell periphery close to VE-cadherin-based cell-cell junctions (Fig. 1a and data not shown), reminiscent of what we have previously described for HUVECs treated with imatinib [27]. Similar results were obtained using antibodies against FA markers paxillin or vinculin (Suppl. Fig. S2a and data not shown). The increase in cell-matrix adhesions and their enrichment at the cell periphery became particularly evident when examining the distribution of integrin α5β1 (Fig. 1e, f). Finally, we tested whether Arg regulates the activation of Rac1, by determining the active fractions of these GTPases in pulldown assays. Basal Rac1 activity was elevated in Arg-depleted HUVECs, explaining the enhanced cell spreading (Fig. 1g).

Taken together, these data show that Arg is a major regulator of integrin-dependent cell-matrix adhesion and spreading in endothelial cells.

### Arg regulates RhoA activation, MLC phosphorylation, and AJ morphology under nonstimulated conditions

**Fig. 2:**
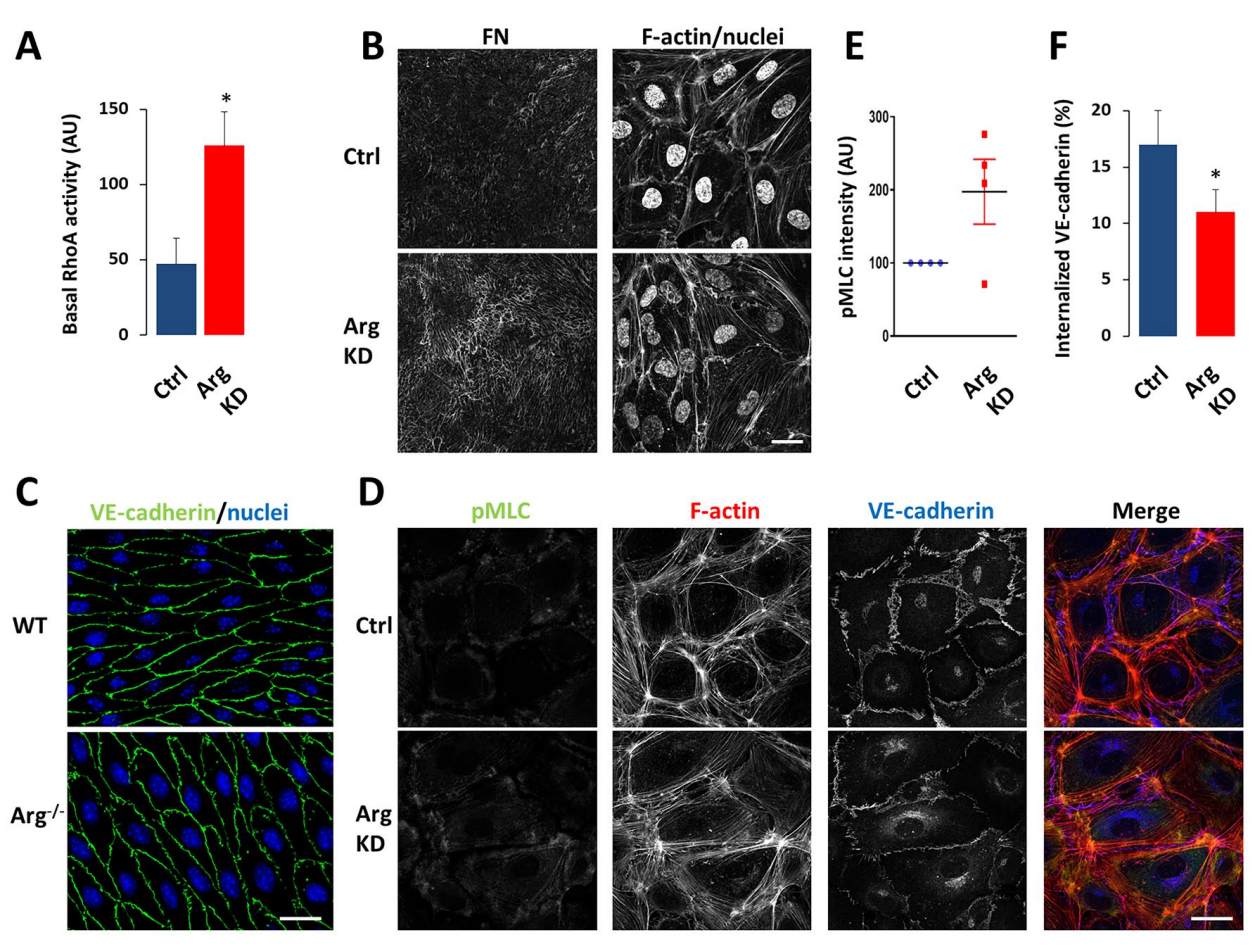
Arg controls RhoA activation, MLC phosphorylation, and AJ morphology in HUVECs. **a** Basal RhoA activity was determined in pulldown assays. Data are means + SEM from *n* = 3. **b** Confocal images of FN fibrils (*left*) and F-actin/DAPI (*right*) in control and Arg-depleted HUVEC monolayers. Bar, 30 μm. **c** Confocal images of VE-cadherin (*green*) and nuclei (*blue*) in femoral arteries of WT and Arg^−/−^ mice. Bar, 10 μm. **d** Representative confocal images of pMLC (*green*), F-actin (*red*), and VE-cadherin (*blue*) in control and Arg-depleted HUVEC monolayers. Bar, 30 μm. **e** Relative pMLC intensity quantified from confocal images. *AU* arbitrary units. **f** Internalization of VE-cadherin in control and Arg-depleted HUVEC monolayers. Data are means + SEM from *n* = 3. *P < 0.05

Since there is a tight balance between Rac1 activity/integrin-dependent cell spreading on the one hand, and RhoA activity/actomyosin contraction on the other hand, we next analyzed whether Arg regulates RhoA activity and signaling. By determining the active fractions in pulldown assays, we observed that basal RhoA activation was significantly enhanced upon Arg silencing (Fig. 2a). This result is consistent with previous studies in fibroblasts [39], and also with the observed increase in cell-matrix adhesions (Fig. 1). Furthermore, Arg-depleted HUVECs assembled a more extensive FN fibril network, which requires integrin α5β1 and RhoA-dependent cytoskeletal contractility (Fig. 2b).

We then assessed the effect of Arg on VE-cadherin-based AJs. The expression of VE-cadherin or β-catenin was not affected by Arg silencing in HUVECs in basal conditions (Suppl. Fig. S3a). Using Arg^−/−^ versus wild-type (WT) littermates obtained from Arg^+/−^ × Arg^+/−^ breedings (Suppl. Fig. S4a), we observed that the expression of VE-cadherin was also unchanged in Arg knockout (Arg^−/−^) mice (Suppl. Fig. S3b). However, AJs in vessels of Arg^−/−^ mice appeared ‘thinner’ and more ‘jagged’ than those in WT mice (Fig. 2c). A similar phenotype was observed in cultured endothelial cells (Fig. 2d), indicating that AJs experienced increased actomyosin-induced pulling forces, consistent with the observed high basal activation of RhoA in Arg-depleted HUVECs. This was further suggested by increased myosin light chain (MLC) monophosphorylation (pSer19) in Arg-depleted cells under basal conditions, which was observed close to VE-cadherin and in particular at tricellular cell-cell junctions (Fig. 2d, e). We also measured VE-cadherin endocytosis in thrombin-stimulated cells, using surface-labeling with biotin followed by capture ELISA as described previously [26, 45–47]. Intriguingly, the uptake of VE-cadherin was significantly reduced in Arg-depleted cells both under basal and stimulated conditions, indicating that Arg promotes VE-cadherin internalization (Fig. 2f). In contrast, the internalization of integrins, as determined using the same assay, was not affected by Arg knockdown (data not shown).

Together, these findings show that Arg regulates RhoA activation and signaling in endothelial cells, as well as the organization of AJs.

### Arg promotes cell retraction in response to thrombin

**Fig. 3:**
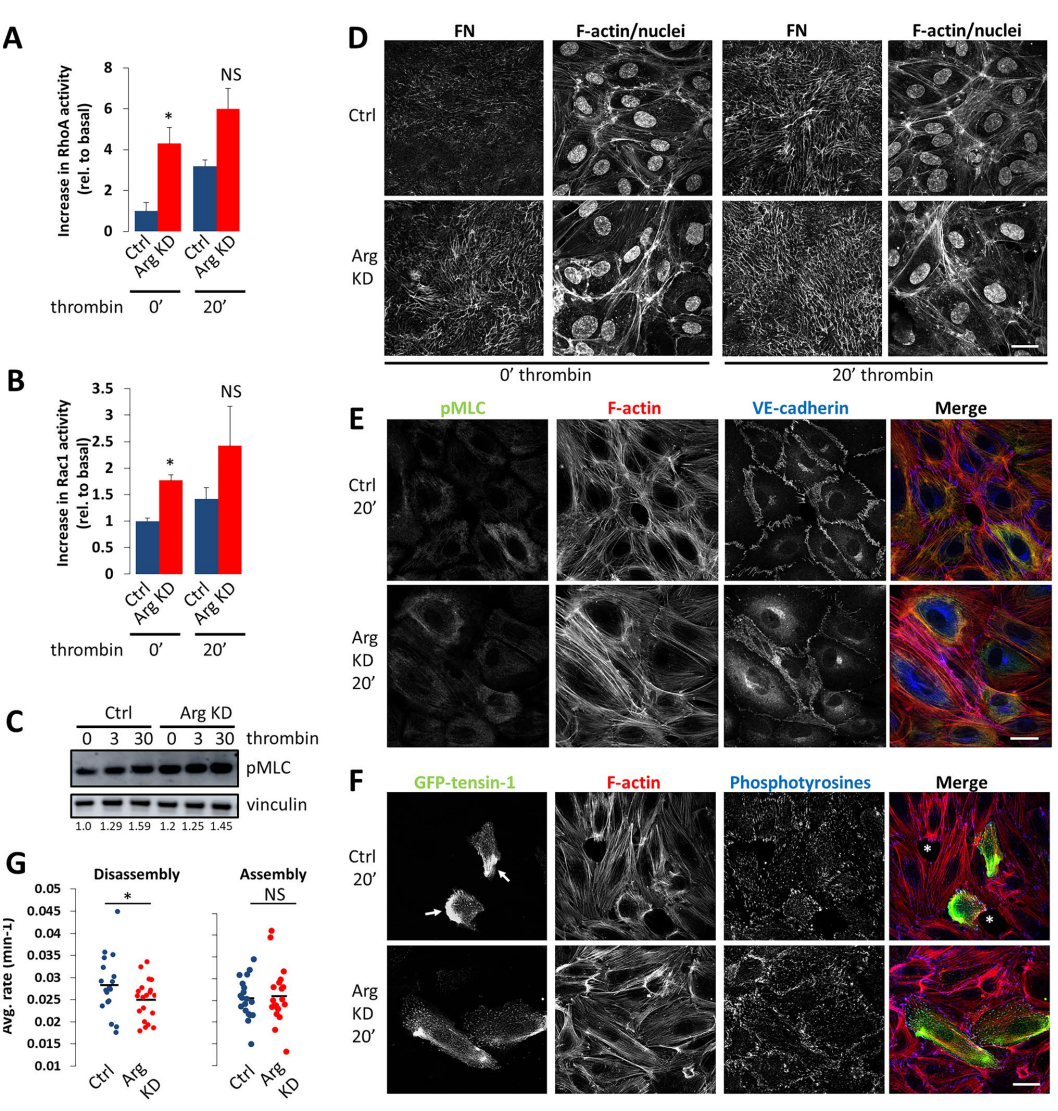
Arg enhances thrombin-induced RhoA activation, redistribution of cell-matrix adhesions, and cell retraction in HUVECs. **a** RhoA activity was determined in pulldown assays at 0 and 20 mins of thrombin stimulation. Data are means + SEM from *n* = 3. **b** Rac1 activity at 0 and 20 mins of thrombin stimulation. Data are means + SEM from *n* = 3. **c** MLC phosphorylation in control and Arg-depleted HUVEC monolayers at the indicated time-points after thrombin addition. Numbers indicate normalized amounts of pMLC, quantified by densitometry and expressed relative to the amount in Ctrl cells at t=0. **d** Confocal images of FN fibrils and F-actin/DAPI in control (*top*) and Arg-depleted HUVEC (*bottom*) monolayers at 0 and 20 mins of thrombin stimulation. Bar, 30 μm. **e** Confocal images of pMLC (*green*), F-actin (*red*), and VE-cadherin (*blue*) in control and Arg-depleted HUVEC monolayers. Bar, 30 μm. **f** Representative confocal images showing the distribution of GFP-tensin-1 (*green*), F-actin (*red*), and phosphotyrosines (*blue*) in control (*top*) and Arg-depleted (*bottom*) HUVEC monolayers 20 mins after thrombin stimulation. Arrows indicate GFP-tensin-1 accumulation, asterisks denote intercellular gaps. Bar, 40 μm. **g** Average assembly and disassembly rate of FBs in control and Arg-depleted HUVECs in thrombin-stimulated monolayers. Each data point represents 1 time-lapse movie. NS, not significant; *P < 0.05

We next investigated how Arg regulates the behavior of endothelial cells when exposed to endothelial barrier-disruptive agents. For this purpose, we chose thrombin, which is a well-studied RhoA activator involved in endothelial barrier disruption. Treatment with thrombin for up to 30 min potently increased RhoA activation in control HUVECs, confirming many other studies [9, 11, 13] (Fig. 3a and data not shown). However, the thrombin-induced increase in RhoA activation was significantly lower in Arg-depleted cells, probably because RhoA activity was already high in basal conditions (Fig. 3a). Rac1 activity was slightly but not significantly increased by thrombin in this time-frame, and was higher in Arg-depleted cells both in the absence and the presence of thrombin (Fig. 3b). In line with the observed differences in RhoA activation, thrombin induced a strong increase in MLC phosphorylation and FN fibrillogenesis in control cells, while Arg-depleted cells had already high levels of phospho-MLC and FN fibrils under basal conditions (Fig. 3c–e). Thrombin-induced increase in phospho-MLC was observed on actin stress fibers and along the entire cell periphery, in particular on circumferential actin filaments (Fig. 3e).

Morphologically, thrombin-induced cell contraction and intercellular gap formation (indicated by asterisks in Fig. 3f) was evident in control, but not in Arg-depleted HUVECs, in which cell spreading remained better preserved (Fig. 3f). The latter appeared not to be due to differences in stress fiber formation (Fig. 3e, f). Arg-depleted cells retained peripheral FAs during thrombin stimulation, as well as centrally located FBs, whereas in control cells a strong, polarized accumulation of tensin was observed, presumably due to RhoA-dependent FB redistribution (indicated by arrows in Fig. 3f).

To analyze the dynamic behavior of FBs in live cells, HUVECs were transfected with GFP-tensin-1, grown to confluency, and the response to thrombin was analyzed by time-lapse microscopy. While thrombin induced clear sliding, accumulation, and eventual disappearance of FBs in control cells, FBs in shArg cells seemed much more resistant against this effect (data not shown). Quantification of the assembly and disassembly rate revealed that whereas the assembly rate was comparable, the disassembly rate of FBs was indeed significantly lower upon Arg silencing, thus reducing their turnover (Fig. 3g).

Together, these results show that Arg promotes cell retraction and redistribution of cell-matrix adhesions in response to thrombin, and that Arg depletion protects against these events.

### Arg promotes endothelial monolayer disruption in response to barrier-disruptive agents

**Fig. 4:**
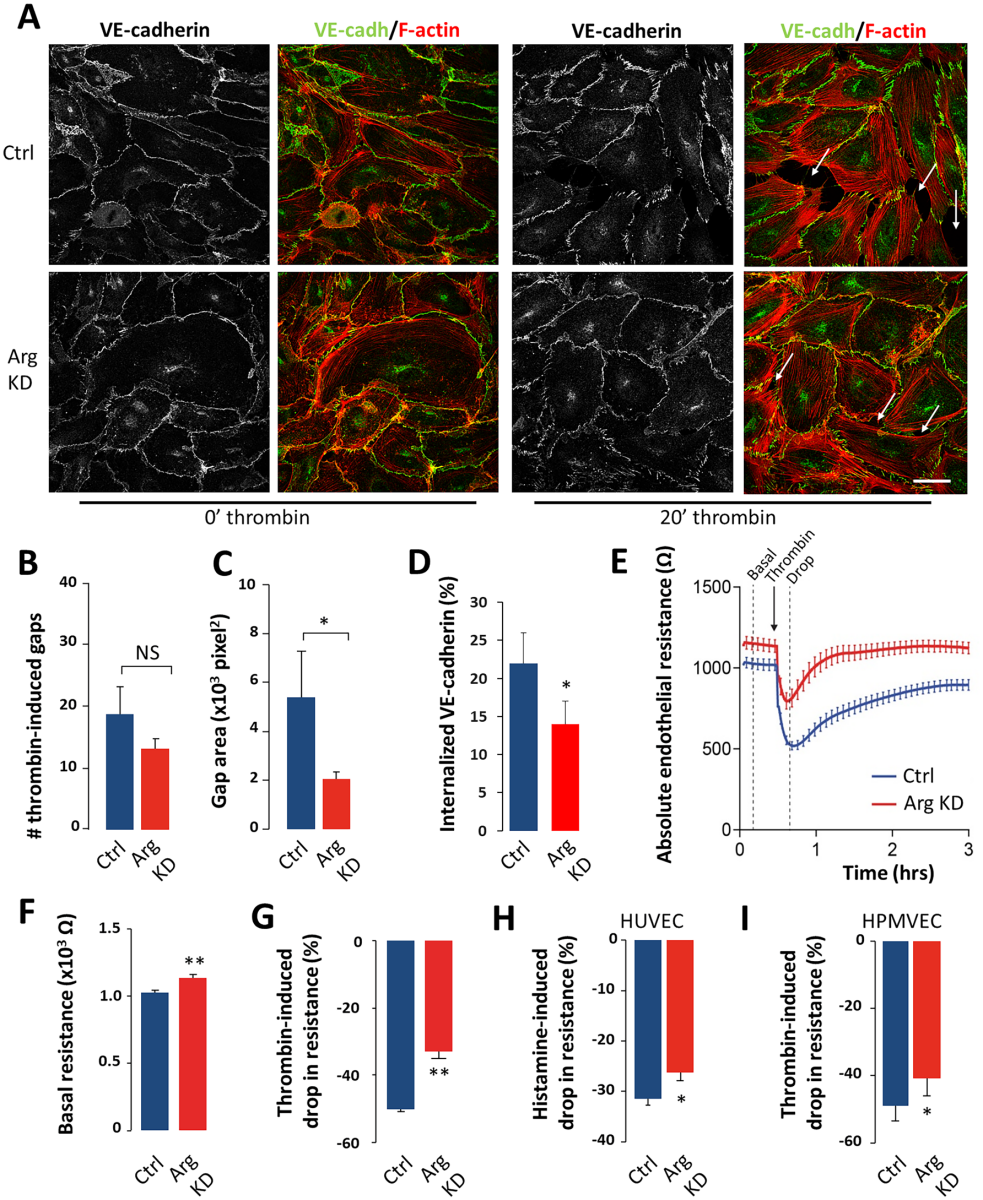
Arg promotes endothelial barrier disruption in vitro. **a** Representative confocal images of VE-cadherin (*green*) and F-actin (*red*), showing AJ morphology in HUVEC monolayers in the absence and the presence of thrombin. Bar, 40 μm. Arrows indicate intercellular gaps. **b** Analysis of thrombin-induced intercellular gap number and (**c**) intercellular gap size. Mean ± SEM of 2-3 images/donor, performed in HUVECs from 3 donors. **d** Internalization of VE-cadherin in HUVEC monolayers stimulated with thrombin. **e** Electrical resistance of confluent HUVEC monolayers before (basal) and after thrombin addition, determined by ECIS. Quantification of **f** basal resistance and **g** thrombin-induced drop in resistance (normalized for differences in basal barrier function), as determined by ECIS. Mean + SEM of *n* = 19 experiments with HUVECs from different donors. **h** Quantification of histamine-induced drop in endothelial resistance in HUVECs. Mean ± SEM of n = 4 experiments with cells from different donors. **i** Quantification of thrombin-induced drop in resistance in HPMVECs. Mean ± SEM of *n* = 4 experiments with cells from different donors. NS, not significant; *P < 0.05; **P < 0.01

To examine the effects of Arg on monolayer integrity in response to barrier-disruptive agents, HUVEC monolayers were stimulated with thrombin for up to 30 min. Thrombin induced pronounced AJ dissociation and intercellular gap formation in control HUVECs (indicated by arrows), which was reduced by Arg depletion (Fig. 4a and data not shown). Of note, Arg depletion predominantly reduced gap size, while gap number was less affected (Fig. 4b, c). Similar to what we observed in non-stimulated conditions (Fig. 2f), VE-cadherin internalization was also significantly decreased in Arg-depleted cells after stimulation with thrombin (Fig. 4d).

We then tested barrier function of HUVEC monolayers using Electrical Cell-substrate Impedance Sensing (ECIS). Downregulation of Arg slightly enhanced basal barrier function, and profoundly attenuated endothelial barrier disruption by thrombin (Fig. 4e–g), in line with the observed reduction in intercellular gap formation (Fig. 4a–c). Similar results were obtained using histamine (Fig. 4h). Moreover, Arg depletion also improved barrier function in human pulmonary microvascular endothelial cells (Fig. 4i). These results indicate that Arg facilitates endothelial barrier disruption in response to a variety of barrier-disruptive factors, and in endothelial cells derived from different vascular beds.

Together, these data show that Arg promotes the disassembly of AJs and intercellular gap formation, thus disrupting endothelial barrier function, and indicate that Arg inhibition can protect against endothelial barrier disruption.

### Arg is activated during inflammation in the endothelium and promotes inflammation-induced vascular leak in vivo

**Fig. 5:**
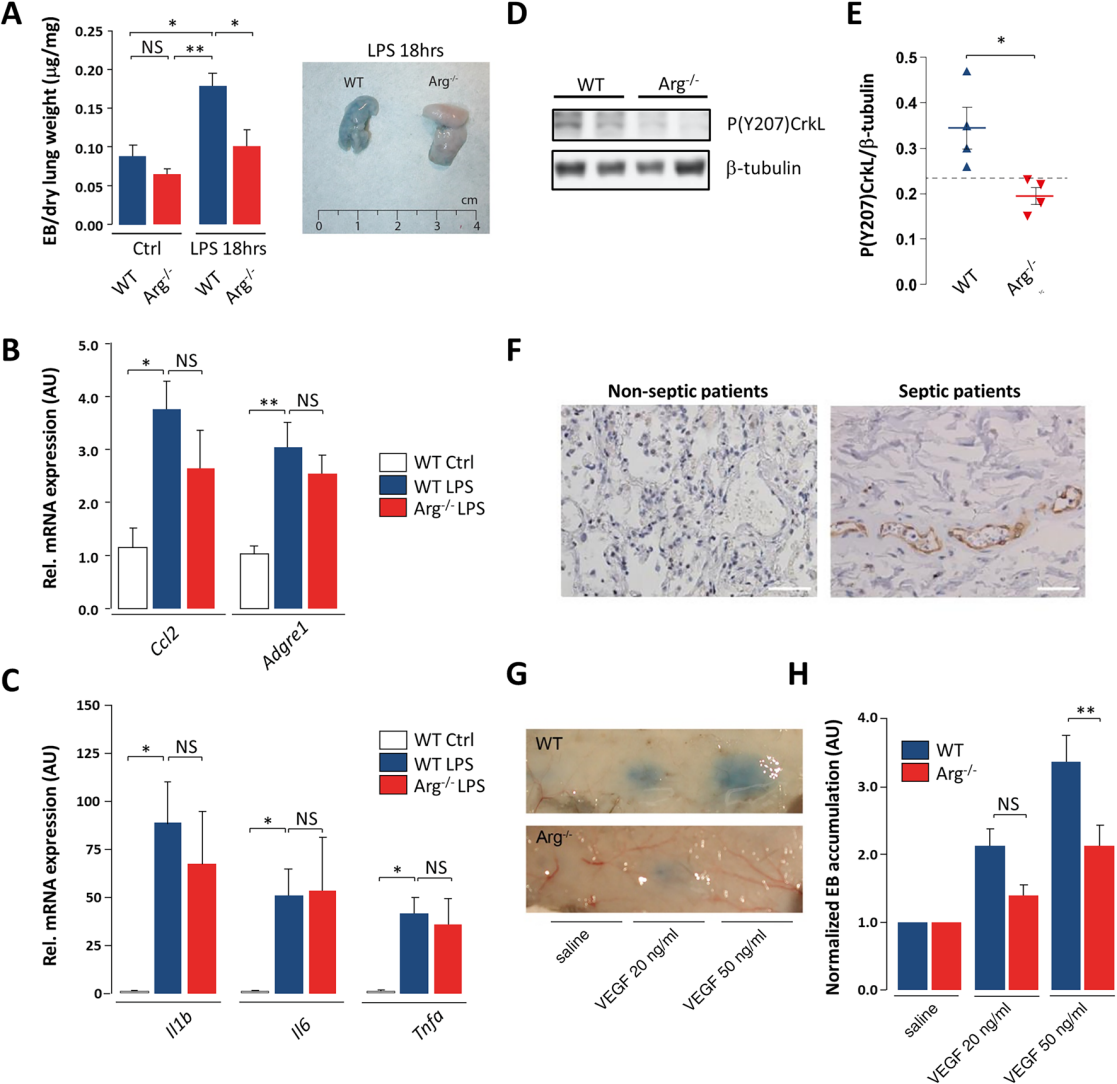
Arg promotes inflammatory vascular leak in vivo. **a** Quantification of extravasated Evans Blue in the lungs of untreated and LPS-treated mice, normalized for dry lung weight (*left*). Mean + SEM of *n* = 4–6 mice per group. Macroscopic images show LPS-induced Evans Blue leakage in the lungs after 18 h (*right*). **b** mRNA levels of macrophage activation markers in lung lysates of WT versus Arg^−/−^ mice exposed to intra-tracheal LPS. **c** mRNA levels of inflammatory cytokine expression in lung lysates of WT versus Arg^−/−^ mice exposed to intra-tracheal LPS. **d** Representative Western blot and (**e**) quantification (*n* = 4) of P(Y207)CrkL, normalized to β-tubulin, in lung tissue of WT and Arg^−/−^ mice. **f** Representative immunohistochemistry images using an antibody against p(Y207)CrkL in paraffin lung slices of 5 septic versus 4 non-septic patients. Brown staining indicates p(Y207)CrkL (predominantly observed in the endothelial layer), blue staining indicates hematoxillin. Bars, 25 µm. **g** Macroscopic images of Evans Blue leakage in the skin after intra-cutaneous administration of PBS or VEGF in WT versus Arg^−/−^ mice. **h** Quantification of VEGF-induced Evans Blue accumulation in the skin, normalized to that in the presence of PBS only. Means +SEM of *n* = 4 mice per group. *AU*, arbitrary units; *NS*, not significant; *P < 0.05; **P < 0.01

We next investigated whether Arg contributes to vascular leak in vivo. Arg^−/−^ mice were apparently healthy and did not show signs of edema or spontaneous bleeding (data not shown). To evaluate the role of Arg in inflammation-induced pulmonary vascular leak, WT and Arg^−/−^ mice were exposed to intra-tracheal instillation of LPS for 18 h, thus mimicking acute lung injury. Vascular leak was measured by injection of Evans Blue (EB) one hr before sacrifice, and the immune response was measured by analyzing expression levels of inflammatory genes by qPCR. In the absence of LPS, no significant differences in EB accumulation were observed between WT and Arg^−/−^ mice (Fig. 5a). In contrast, instillation of LPS resulted in an increase in pulmonary vascular leak in WT mice, which was significantly reduced in Arg^−/−^ mice (Fig. 5a). We observed a comparable immune response in Arg^−/−^ mice versus WT mice upon LPS exposure, as measured by markers of macrophage activation and cytokine production (Fig. 5b, c). In addition, there was no significant difference in the numbers of extravasated cells found in the broncho-alveolar lavage fluid from WT versus Arg^−/−^ mice (Suppl. Fig. S4b).

Together, these results indicate that Arg depletion protects against inflammation-induced endothelial barrier disruption, while the inflammatory response and leukocyte extravasation occur normally.

In WT mice, vascular leak was paralleled by phosphorylation of CrkL at Y207, a well-known substrate of Abl kinases [41] (Fig. 5d, e). In contrast, strongly reduced LPS-induced CrkL phosphorylation at this residue was observed in Arg^−/−^ mice, indicating that Arg is activated in the lung during inflammatory vascular leak (Fig. 5d, e). While it is possible that this result reflects the combined deletion of Arg in multiple cell types, we also investigated whether Arg-dependent (Y207)CrkL phosphorylation occurs in response to a barrier-disruptive stimulus in HUVECs. Indeed, thrombin stimulation caused an increase in the levels of p(Y207)CrkL, which was reduced by Arg depletion (Suppl. Fig. S4c). As a proof of concept of Arg activation in clinical disease associated with vascular leak, we performed immunohistochemistry for phosphorylation of CrkL on lung slices from critically ill, septic versus non-septic patients. Intriguingly, positive staining for p(Y207)CrkL was found more often in the lungs of septic patients than in non-septic patients, predominantly in the endothelium of microvessels, suggesting that Arg activation in lung endothelium occurs during sepsis (Fig. 5f, Suppl. Table S1, Suppl. Fig. S5). Finally, we also tested the effects of Arg on vascular endothelial growth factor (VEGF)-induced vascular leak. For this purpose, WT and Arg^−/−^ mice were injected with VEGF or saline in the back skin after systemic administration of EB. Importantly, Arg^−/−^ mice were protected against VEGF-induced vascular leak in the skin, while no differences between WT and Arg^−/−^ mice were observed upon saline injection (Fig. 5g, h).

Together, these data indicate that Arg is a regulator of endothelial barrier disruption and vascular leak in vivo, and determines the endothelial response to a variety of barrier-disruptive stimuli.

## Discussion

**Fig. 6:**
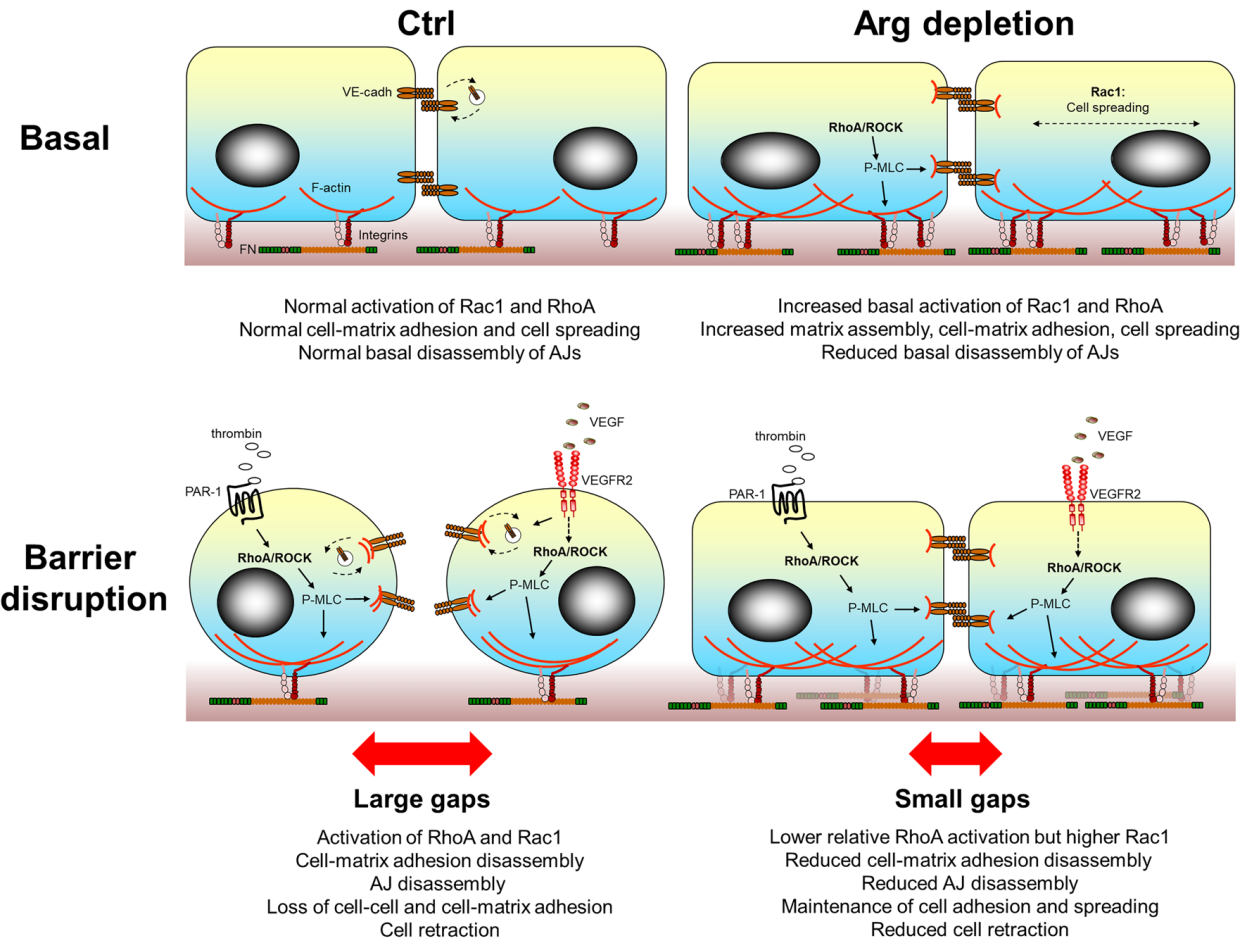
Model summarizing the results described in this study. In resting endothelium, Arg depletion leads to increased basal activation of both RhoA and Rac1, thus increasing matrix assembly and cell-matrix adhesion and spreading, while the basal disassembly of AJs is reduced. Upon stimulation with a barrier-disruptive agent (thrombin, histamine and to a lesser extent VEGF), increased RhoA activation leads to cytoskeletal contractility-mediated loss of cell-cell adhesion and cell retraction in control cells. In contrast, RhoA activation and contractility in Arg-depleted cells are balanced by higher Rac activation, and lower AJ disassembly, thus preserving cell-cell and cell-matrix adhesion and counteracting retraction.

Here, we identify the Abelson-family kinase Arg/Abl2 as a key regulator of endothelial barrier function. We show that knockdown of Arg expression in endothelial cells in vitro induces a variety of effects on cell morphology, Rho GTPase activity, integrin-dependent cell-matrix adhesions, and VE-cadherin-based cell-cell junctions (Fig. 6). Moreover, this is the first study to demonstrate the non-redundant function of Arg in endothelial barrier regulation in vivo, and to directly demonstrate its involvement in clinical conditions like pulmonary vascular leak and acute lung injury.

Arg depletion enhanced the number of integrin-based cell-matrix adhesions, in particular at the cell periphery, in line with previous observations using imatinib [27, 32]. It has been shown previously that Arg overexpression can induce phosphorylation of the NPxY motif in the integrin β1 cytoplasmic tail [42]. However, we only detected Y783 phosphorylation in the presence of phosphatase inhibitors, indicating that it is probably weak and/or transient. More importantly, Y783 phosphorylation was not abrogated by Arg depletion, suggesting that Arg is not required for this event in endothelial cells. Moreover, integrin activation and internalization, which are regulated by this motif, were normal in Arg-depleted HUVECs. The observed increase in cell-matrix adhesions is most likely due to the strong increase in RhoA activity upon knockdown of Arg expression. The RhoA/Rho kinase pathway enhances MLC phosphorylation by reducing its dephosphorylation, and the resulting cytoskeletal contractility promotes the stability of cell-matrix adhesions including FAs and FBs, as well as the associated integrin α5β1-dependent organization of FN into fibrils [48]. Indeed, we observed increased basal MLC phosphorylation and FN fibrillogenesis, as well as decreased FB turnover in Arg-depleted cells. Our findings are in line with a previous study in fibroblasts, in which it was found that Arg phosphorylates p190 RhoGAP, which inhibits Rho activity [39]. Intriguingly, increased cytoskeletal contractility can disrupt cell-cell junctions, and although the ‘jagged’ appearance of VE-cadherin in the absence of Arg suggests that there is indeed increased tension on AJs, it did not perturb monolayer integrity and barrier function. Moreover, the increase in contractility did not abolish cell spreading, which was in fact enhanced. The increase in cell spreading is likely due to the effects of the well-established Arg substrate CrkL, which stimulates Rac1 activation and cell spreading but whose phosphorylation at Y207 by Abl tyrosine kinases negatively regulates these events [41, 49, 50]. Consistent with this hypothesis is that we observed reduced p(Y207)CrkL in HUVECs upon Arg depletion, while Rac1 activity was increased. There is ample evidence that Rac1 counteracts Rho-dependent contractility, and that regulation of cell size and cell spreading by Rac1 promotes the stability of AJs, thus preserving endothelial barrier function [7, 8, 10]. Furthermore, Rac1-stimulated cell spreading enhances the formation and stability of peripheral cell-matrix adhesions, which in conjunction with cell-cell junctions maintain the spread endothelial cell shape required for an intact monolayer, thus forming a tethering force to prevent cell retraction. Additionally, Arg may also determine the localization of active RhoA and/or phosphorylated MLC. While the results presented here show effects of Arg on global RhoA activity, future studies should address how Arg affects local activation of different subcellular RhoA pools, for instance using a RhoA biosensor [51]. Interestingly, we observed phosphorylated MLC predominantly close to tricellular cell-cell junctions under basal conditions, while thrombin stimulated increased phospho-MLC in the cell periphery and its association with circumferential actin bundles, consistent with earlier observations that phosphorylated MLC interacts with the cortical actin ring and regulates cell-cell junction integrity [52, 53]. Of note, in a previous study in epithelial cells, the combined knockdown of both c-Abl and Arg enhanced the activity of RhoA but not that of Rac1, and in this system the increased cytoskeletal tension did result in the disruption of E-cadherin-based AJs [33]. While these data further underscore the importance of Rac1 activation to counteract RhoA-dependent contractility in the control of cell-cell junctions, it remains to be established whether there are cell-type-specific differences in Rac1 regulation by Abelson kinases, or whether this is differentially regulated by c-Abl and Arg.

Importantly, we show that in the presence of c-Abl expression, the depletion of Arg is sufficient to preserve the barrier function of the endothelium, even after challenge with barrier-disruptive agents. Upon activation of RhoA with thrombin, the relative increase in RhoA activity and MLC phosphorylation were smaller in Arg-depleted cells, probably because the basal levels are already almost maximal. Furthermore, thrombin induced less cell retraction and intercellular gap formation in Arg-depleted cells, thus rendering the cells more refractory against barrier disruption. Together, these data suggest that Arg depletion reduces the dynamic behavior of cell-cell and cell-matrix adhesions and improves anchorage of the plasma membrane at the cell periphery (Fig. 6). The dynamic regulation of peripheral cell adhesions contributes to cell retraction and gap formation during endothelial exposure to barrier-disruptive agents, as observed in inflammation, hence anchoring of the cell periphery in Arg-depleted endothelial cells protects against barrier disruption.

Because Arg knockout mice are healthy and show no obvious defects, long-term absence of Arg is well-tolerated in vivo. Importantly, induction of inflammatory markers and cell counts in the broncho-alveolar lavage fluid were comparable in Arg^−/−^ mice versus WT mice upon LPS exposure, corroborating previous reports that vascular leak and leukocyte extravasation are separately regulated processes [51, 54]. These findings indicate that Arg inhibition can be harbored as treatment strategy to target vascular leakage, while leaving the immune response intact [55]. Finally, we also observed that Arg deficiency inhibited VEGF-induced vascular leak in the skin. While Arg may regulate the response to VEGF at several levels, which will be important to analyze in more detail in future studies, this finding may suggest that Arg is central to vascular leak induced by a variety of different factors. This is further suggested by our observations that Arg knockdown protected against barrier disruption induced by both thrombin and histamine in vitro. Therefore, we propose that Arg is a central and non-redundant regulator of endothelial barrier disruption in a variety of conditions and that inhibition of Arg is a suitable strategy for the treatment of clinical syndromes characterized by vascular leak, including sepsis, ARDS, and COVID-19. This is of utmost importance for critically ill patients with respiratory failure as a result of pulmonary edema, because currently no drugs are available to reverse endothelial barrier disruption under these conditions. We and others have previously shown that pharmacological inhibition of Arg by imatinib protects against leakage-induced pulmonary edema in preclinical studies [27], as well as in patients [28, 29, 31]. Moreover, a clinical trial has recently been completed by us (EudraCT # 2020-001236-10), to establish whether imatinib also protects against respiratory failure in patients suffering from COVID-19.

Of note, imatinib inhibits c-Abl in addition to Arg, which is important because c-Abl also promotes endothelial barrier disruption [35–38]. Whereas previous studies have demonstrated the effect of c-Abl knockout [34–36] and have addressed the redundant roles of c-Abl and Arg in endothelial barrier regulation [35], our study for the first time points towards a specific and non-redundant function of Arg in vivo. Together with the previously mentioned in vivo studies [34–36], it becomes apparent that while Arg and c-Abl are both involved in endothelial barrier regulation, they probably have complementary and distinct functions. Future studies are required to elucidate the unique roles of c-Abl and Arg in barrier regulation. Because imatinib also inhibits additional tyrosine kinases, including the platelet derived growth factor receptors, development of second-generation inhibitors with increased specificity towards Abl/Arg would be preferred. Other tyrosine kinase inhibitors exist with overlapping and unique specificities, and we have recently characterized the effects of bosutinib on endothelial barrier function [56]. Bosutinib inhibits Arg as well as MAP4K4, which phosphorylates ERM proteins and thereby regulates endothelial cell adhesion [56, 57]. Thus, multiple kinases work in concert to effectuate the dynamics of endothelial cell adhesions, and it will be important in the future to further delineate the effects of each for the optimal treatment of vascular leak.

## Materials and methods

### Antibodies, plasmids and other materials

The following antibodies were used: anti-c-Abl (#2862), anti-β-tubulin (#2128), anti-pSer19 myosin light chain (#3671), anti-CrkL (#3182), and anti-p(Y207)CrkL (#3181) from Cell Signaling Technologies, anti-VE-cadherin (clone C19 from Santa Cruz and clone 55-7H1 from BD Pharmingen), anti-β-catenin (Sigma Aldrich), anti-α5 integrin (clone VC5 from BD Pharmingen; clone MAB1999 from Chemicon), anti-Arg (NBP1-18875 from Novus Biologicals), anti-integrin β1 (clone 9EG7 from BD Pharmingen; clone TS2/16 from the Developmental Studies Hybridoma Bank; clone P5D2 from Abcam; clone EP974(2)Y against phosphorylated Y783 from Abcam), actin (clone AC-40) and phosphotyrosines (clone 4G10 from Sigma-Aldrich), FN (clone 10), Rac1 (clone 102), and paxillin (clone 165) were from Transduction laboratories, RhoA (clone 67B9 from Cell Signaling). GFP-tensin-1 was a kind gift from Dr. K. Yamada (National Institute of Health, Bethesda, MD), Rhotekin-RBD and PAK-CRIB peptides were home-made. Phalloidin was from Thermo Fischer, puromycin, human thrombin, human FN, Na3VO4, and *E. coli*-derived LPS were purchased from Sigma-Aldrich, VEGF from Invitrogen, and Col-I was from Vitrogen. TRITC-, FITC-, and Cy5-conjugated secondary antibodies, and DAPI were from Molecular Probes, Fugene was from Promega, and HRP-conjugated secondary antibodies were from Amersham. Primers were purchased from Integrated DNA Technologies, Inc. (sequences shown in Supplementary Table S2).

### In vivo measurement of vascular leakage

Arg knockout mice with mixed 129/SvJ × C57BL/6J background were kindly provided by Dr. A. Koleske (Yale University, New Haven, Connecticut). Heterozygous mice were bred to obtain homozygous knockout mice and wild-type littermates for experiments. Genotyping was performed with RT-PCR (primer sequences shown in Supplementary Table S2). For experiments, 10–24 weeks old mice (20–40 gr) were used, male and female in equal numbers per group.

For measurement of vascular leakage in the skin, mice were anesthetized by intraperitoneal injection with fentanyl, midazolam and acepromazine [58]. EB dye (0.5% in PBS, 150 µL) was injected via the tail vein. Mice were shaved after 30 min, and saline or VEGF (20 or 50 ng, dissolved in saline) was injected intra-dermally in the back skin. Mice were sacrificed 30 min later and the back skin was isolated. Pictures were taken and circular skin patches (ø 8mm) from the injection sites were incubated in formamide for 48 h. Concentrations of EB and hemoglobin were measured spectrophotometrically at 610 and 740 nm, respectively.

For measurement of vascular leakage in the lungs, mice were anesthetized with isoflurane (4% v/v in air during induction and 1.5%–2% maintenance) and oxygen 0.5 L/min. The trachea was exposed by small incision of the skin, and oro-tracheal canulation was performed under view, followed by intra-tracheal administration of LPS (2.5 mg/kg) dissolved in 50 µL saline, or saline only. Mice were kept upright and mildly agitated to ensure that LPS or saline reached peripheral parts of the lungs. After 17 h, mice received 100 µL 1% EB via tail vein injection, which was allowed to circulate for 1 h, after which mice were sacrificed under anesthesia. EB was extracted from the right lung by incubating in 300 µL formamide at 55 °C. After 48 h the lungs were removed; the remaining formamide was centrifuged (13,500 rpm for 5 min) and analyzed spectrophotometrically. The corrected EB absorbance was calculated by the following formula: OD610-[1.426 × OD740 + 0.03], and compared to an EB standard. The lungs were air-dried at 90 °C to determine dry weight, and vascular leak was represented as EB/dry lung weight (µg/mg). The left lung was snap-frozen for tissue analysis. All animal experiments were approved by the local ethical committee for animal welfare, according to national and international guidelines.

### Cell culture, transfections, lentiviral transduction, and RNA interference

Primary HUVECs pooled from 3 to 5 individual donors were purchased from Lonza (C2519A) and were cultured in endothelial growth medium-2 (Promocell, C-22011), supplemented with 2 mM L-glutamine (Sigma-Aldrich) and 1 U/ml penicillin/streptomycin (Sigma-Aldrich). HUVECs were used between passages 3 and 6 and routinely passaged on cell culture flasks coated with 0.1% (w/v) gelatin (Sigma-Aldrich). Human pulmonary microvascular endothelial cells were freshly isolated and cultured as described before [59]. Human embryonic kidney (HEK) 293T cells (ATCC, CRL-3216) were maintained in Dulbecco’s modified Eagle medium (DMEM) (Thermo Fisher Scientific) containing 4.5 g/l D-glucose, 2 mM L-glutamine, 10% (v/v) fetal bovine serum (Bodinco), 1 mM sodium pyruvate (Thermo Fisher Scientific), and 1 U/ml penicillin/streptomycin. All cells were maintained at 37°C in a humidified atmosphere containing 5% CO2.

For RNA interference we used shRNAs cloned into pLKO.1 (sequences shown in Supplementary Table S3) from the TRC Mission Library (a generous gift from Roderick Beijersbergen, Robotics and Screening Center, Netherlands Cancer Institute, Amsterdam). To produce lentiviral particles containing shRNAs, HEK293T cells were transfected using TransIT-LT1 transfection reagent (Mirus Bio) according to the manufacturer’s protocol. Supernatant was harvested 48 and 72 h after transfection, centrifuged, filtered over a 0.45 μm pore filter, aliquoted and stored at −80 °C. HUVECs were lentivirally transduced with either a pool of shRNAs, or with a scrambled sequence in pLKO.1 as a control. Positive cells were selected during 3 days using 1 μg/ml puromycin. Alternatively, siRNAs were obtained from Dharmacon (Waltham, MA) (sequences shown in Supplementary Table S3), and transiently transfected into HUVECs using DharmaFECT transfection reagent Type 1 (Dharmacon) according to the manufacturer’s instructions. Cells received fresh medium 24 h later, and experiments were performed 48 h after transfection. Knockdown efficiency was evaluated in each experiment, either by Western blotting or by qPCR analysis, and similar results were obtained using siRNAs or shRNAs (data not shown).

Transient transfections of GFP-tensin-1 were performed by electroporation using the Amaxa nucleofector as described previously [26].

### Flow cytometry

HUVECs were treated as indicated, detached with trypsin, and washed twice in PBS containing 2% FCS. Cells were then stained with primary antibodies for 1 h on ice, washed twice, followed by incubation with secondary antibodies for 45 min on ice and washed twice. Cells were analyzed on a Canto-II flow cytometer (BD Immunocytometry Systems) equipped with FACSDiva software.

### Cell lysis and western blotting

Cells were washed in ice-cold PBS and lysed on ice in RIPA buffer (25 mM Tris/HCl pH 7.6, 150 mM NaCl, 1% NP-40, 0.5% sodium deoxycholate, 0.1% SDS), supplemented with protease inhibitors and phosphatase inhibitors (Sigma-Aldrich). Cell lysates were centrifuged at 13,000×*g*, heated at 95 °C in SDS sample buffer (50 mM Tris-HCl pH 6.8, 2% SDS, 10% glycerol, 1% β-mercaptoethanol, 12.5 mM EDTA, 0.02 % bromophenol blue), and proteins were resolved by SDS-PAGE, after which they were transferred to PVDF membranes (Millipore).

For protein analysis of mouse organ tissue, organs were snap-frozen after sacrificing the animal. Snap-frozen tissue was processed to tissue lysates by mincing 50 mg of snap-frozen tissue in 500 µL of RIPA buffer with phosphatase inhibitors and protease inhibitors. Protein concentrations were adjusted to 2 µg/µL using Bradford reagents, resolved by SDS-PAGE, and transferred to PVDF membranes for Western blotting.

Aspecific binding was blocked using 5% (w/v) milk (Campina) in TBST (150 mM NaCl, 10 mM Tris, 0.1% Tween 20, pH 8.0) for 30 min, followed by incubation with primary antibodies (o/n at 4°C) and with secondary antibodies for 1 h at RT. Membranes were washed 3× with TBST after each step. Proteins were visualized using ECL reagent (Thermo Fisher Scientific) or the SuperSignal system (Pierce Chemical Co.), on light-sensitive films (Fuji Film) using a film processor (Konica Minolta, SRX-101A). Where indicated, quantification of band intensity was performed by densitometry using Fiji/ImageJ. Band intensity of the protein of interest was corrected to that of the corresponding loading control (β-tubulin or vinculin).

### Rac1 and RhoA activity assays

Rac1 and RhoA assays were performed as previously described [60]. Briefly, cells were seeded in 10cm dishes coated with FN (5 μg/ml) at 70–80% confluency. HUVECs were stimulated with thrombin (1 U/ml in serum-free medium) the next day for the indicated time-points and then lysed. All cells were washed with ice-cold PBS prior to lysis in buffer containing 25 mM Tris-HCl pH 7.2, 150 mM NaCl, 10 mM MgCl2, 1% NP-40, 5% glycerol, and protease inhibitors. Lysates were centrifuged for 5 min, 14000×*g* at 4°C, and incubated with bacterially produced GST-Rhotekin-RBD beads (RhoA activation assay) for ≥ 1 h at 4 °C. After incubation, samples were centrifuged for 20 sec, 5000×*g* at 4°C, GST-Rhotekin-RBD beads were placed on ice, and supernatants were incubated for 30 min with 30 μg of a biotinylated PAK1-CRIB peptide (Rac1 activation assay) coupled to streptavidin agarose beads. Subsequently, all beads were washed 5 times with lysis buffer, boiled in SDS sample buffer, and analyzed by Western blotting. Bands of pulldowns as well as total cell lysates were quantified by densitometry using Fiji/ImageJ. Results are expressed as the ratio active/total GTPase.

### Internalization assays and capture-ELISA

Internalization assays were performed essentially as described earlier with some modifications [26, 45–47]. Briefly, cells were treated as indicated, transferred to ice, washed twice in ice-cold PBS and surface-labeled at 4 °C with 133 μg/ml NHS-SS-biotin for 60 min. Cells were washed 3 times with ice-cold PBS before transfer to serum-free medium at 37 °C in the presence of 0.6 mM primaquine (Sigma-Aldrich) to block recycling, after which they were washed twice with ice-cold PBS. Remaining cell-surface biotin was removed by reduction with 3.89 mg/ml MesNa (Merck) and 0.375 μl/ml 30% H2O2 (Merck). After washing in PBS, the reduction was quenched with 3.45 mg/ml Iodoacetamide (Sigma-Aldrich) for 60 min at 4C, whereafter the cells were lysed in 200 mM NaCl, 75 mM Tris, 7.5 mM EDTA, 7.5 mM EGTA, 2.25% NP-40, 100 μM Na3VO3, and protease inhibitor cocktail (Sigma-Aldrich).

Maxisorb 96-well plates (Life Technologies) were coated o/n with 5 μg/ml anti-integrin α 5 (Clone VC5), anti-integrin β1 (Clone P5D2), or anti-VE-cadherin (Clone 55-7H1) antibodies in 0.05 M Na2CO3, pH 9.6 at 4 °C, and then blocked for 1 h at RT in PBST with 5% BSA. Plates were incubated with cell lysates (o/n at 4°C), washed with PBST, and incubated with HRP-streptavidin in PBST with 0.1% BSA for 1 hr at 4 °C. After washing, biotinylated proteins were detected with ortho-phenylenediamine (Sigma-Aldrich) in a buffer containing 25.4 mM Na2HPO4, 12.3 mM citric acid (pH 5.5) and 0.003% H2O2. The reaction was terminated with 8 M H2SO4 and absorbance was read at 490 nm.

### Live imaging of fibrillar adhesions and analysis

HUVECs were transduced with shRNAs and selected with puromycin, whereafter they were transfected with GFP-tensin-1 and grown to confluency on Nunc Lab-Tek Chambered #1.0 Borosilicate coverglass systems (Thermo Fisher Scientific). Cells were stimulated with thrombin the next day for the indicated time, and time-lapse sequences were recorded on a Zeiss Observer Z1 microscope (40× magnification, 60 s intervals). Movies were generated with Fiji/ImageJ, and the assembly and disassembly rate of fibrillar adhesions were determined using open-source software (http://gomezlab.bme.unc.edu/tools) essentially as described previously [61] with the following specifications: imaging frequency 1, detection threshold 2, min adhesion size 5, min FA phase length 10, min FAAI ratio 3.

### qPCR

For analysis of relative mRNA expression levels in transduced HUVECs, total RNA was isolated using the RNeasy kit (Qiagen) according to the manufacturer’s instructions. After reverse transcription to cDNA using the SuperScript III First-Strand Synthesis System (Thermo Fisher Scientific), qPCR was performed with the SensiFAST SYBR No-ROX kit (Bioline) and the indicated primers (Supplementary Table S2). The PCR was performed and analyzed either on a LightCycler PCR system (Roche) or a StepOnePlus system (Applied Biosystems). Duplicate reactions were performed for each gene.

For mRNA analysis of mouse lung tissue, lungs were snap-frozen in liquid nitrogen directly after sacrificing the animal. RNA was isolated from 30 mg of snap-frozen lung tissue, using the RNeasy Minikit (Qiagen). Isolated mRNA was used for cDNA synthesis, using iScript reaction mix and iScript reverse transcriptase. cDNA was used for qPCR with the indicated primers (Supplementary Table S2). Reference genes for cultured endothelial cells included *GAPDH* or *TUBB2*, while *Rps15* was used as a reference gene for qPCR analysis of mouse tissue.

### Confocal microscopy and quantifications

HUVECs on glass coverslips were treated as indicated and then fixed with 4% paraformaldehyde (Merck) in PBS containing 1 mM CaCl2 and 0.5 mM MgCl2 (PBS++) for 10 min, and permeabilized with 0.4% Triton X-100 (Sigma-Aldrich) in PBS++ for 5 min. Aspecific antibody binding was prevented by blocking with 2% BSA Fraction V (Sigma-Aldrich) in PBS++ for 15 min. Following incubation with the indicated primary antibodies, coverslips were washed with 0.5% BSA Fraction V in PBS++ and antibody binding was visualized using secondary antibodies. Coverslips were washed and subsequently mounted in 10% Mowiol, 2.5% Dabco, 25% glycerol, pH 8.5. Image acquisition was performed on a Leica SP8 confocal microscope using a 63× oil immersion objective, after which images were processed using Leica Application Suite X and Fiji/ImageJ (version 1.52e) software.

The number of peripheral adhesions relative to the cell as a whole was quantified using Fiji/ImageJ essentially as described earlier with some modifications [39]. In short, images were converted to 8-bit grayscale, foreground/background colors were inverted, and threshold-adjusted. Individual cell outlines were generated using the freehand selection tool. The “analyze particles” function was used to select and measure the peripheral area of fluorescence within the freehand selection. The particle areas for the peripheral selection were summed and divided by the total freehand selection area to obtain the relative density at the periphery. Individual selection densities from 20 cells were averaged to obtain the density values per cell. To quantify thrombin-induced intercellular gap formation from confocal images, images were converted to binary images, in which staining-negative areas were considered as being a gap. Gap number and size were determined using the ImageJ particle analysis tool.

Femoral artery segments (1–2 mm) were freshly isolated from sacrificed mice and mounted to glass canulae. After saline perfusion under pressure, vessels were fixed with 4% paraformaldehyde (Sigma Aldrich) for 15 min at room temperature. Fixed vessel segment were cut in the length, opened and mounted to a silicon underground with tungsten wire at the four corners. Subsequently segments were washed with saline and stained with a primary antibody against VE-cadherin (rabbit, 1:200) overnight at 4 °C. After washing, cells were incubated with FITC- and Cy3-labeled secondary antibodies (anti-mouse and anti-rabbit, 1:100) (Invitrogen, Paisly, United Kingdom). Finally, vessel segments were mounted on glass coverslips with Vectashield mounting medium, containing DAPI (Vector Laboratories Inc, Burlingham, CA). Imaging was performed as described for cultured endothelial cells.

### Immunohistochemistry of human lung tissue

Paraffin-embedded lung tissue of septic and critically ill, non-septic patients obtained by autopsy was provided by the Department of Pathology of our hospital (Amsterdam University Medical Center, location VUmc, Amsterdam, The Netherlands). Paraffin slices (5µm) were de-paraffinized with xylene and incubated with H_2_O_2_ to block endogenous peroxidase. After antigen retrieval (TRIS/EDTA 10 mM pH 9.0, 15 min at 100 °C), slices were incubated with primary antibody against p(Y207)CrkL (o/n at 4 °C). Detection of primary antibody was performed with Powervision® (Immunologic, Duiven, The Netherlands), according to the manufacturers protocol. After counterstaining with hematoxylin, slices were evaluated with a Leica DMRB light microscope (Leica Microsystems, Wetzlar, Germany) at 20x (air, NA 0.40) and 40x (air, NA 0.65) magnification. For imaging, a Nikon D50 digital camera (Nikon Corporation, Tokyo, Japan) was used.

### Endothelial barrier function measurements

Endothelial barrier function was measured by Electrical Cell-substrate Impedance Sensing (ECIS). Confluent cells were seeded in 1:1 density (5 × 10^4^ cells/cm^2^) on gelatin-coated ECIS arrays, containing 8 wells with 10 gold electrodes/well (Applied Biophysics, Troy, NY). Culture medium was renewed 24 h after seeding, while experiments were performed 48 h after seeding. For thrombin stimulation, cells were incubated in serum-free medium containing 1% human serum albumin (HSA). After 60–90 min of preincubation, thrombin (1 U/ml) or histamine (10 μM) was added to the wells.

### Cell adhesion and cell spreading assays

Plates were coated with Col-I (3 μg/ml, 5 min) or FN (5 μg/ml, 90 min) at 37 °C. Thereafter, they were washed twice with PBS, blocked with 2% BSA/PBS for 30 min at 37 °C, and washed with PBS. For adhesion assays, subconfluent cells were trypsinized, resuspended in serum-free medium, and then seeded in 96-well plates coated with Col-I or FN at a density of 7 × 10^4^ cells per well. After 10 min at 37 °C, non-adherent cells were washed away with PBS. The adherent cells were fixed in 4% PFA, washed twice with H2O, stained for 10 min with crystal violet, washed twice with H2O, and then lysed in 2% SDS. Absorbance was measured at 490 nm on a microplate reader. Background values (binding to BSA-coated wells) were subtracted from all other values, and the number of adherent cells was normalized to that of control cells.

For cell spreading, cells were seeded in serum-free medium on 24-well plates coated with Col-I or FN, and cell spreading was allowed for 3 h. Cells were then photographed on a Widefield CCD system using 10× and 20× dry lens objectives (Carl Zeiss MicroImaging, Inc.). Cell area and elongation index (length/width) were determined using Fiji/ImageJ.

### Statistical analysis

Statistical analysis was performed using one-way ANOVA for multiple comparisons and unpaired *t* tests for comparisons between two conditions, unless stated otherwise. Throughout the paper, statistical significance is indicated by *(P < 0.05) and **(P < 0.01).

## Supplementary Information

Below is the link to the electronic supplementary material.

Suppl Fig. S1. Selective knockdown of Arg but not c-Abl in HUVECs. (A) Relative mRNA levels of Arg (left) and c-Abl (right) in HUVECs expressing scrambled sequences (Ctrl) or short hairpins against Arg. Quantifications are means + SEM from n=6, normalized to GAPDH. (B) Western blot showing expression of Arg and c-Abl in HUVECs. GAPDH was used as a loading control. Experiment is representative of n=5 (TIF 89 KB)

Suppl Fig. S2. Arg controls organization of cell-matrix adhesions, cell spreading, and β1 integrin expression in HUVECs. (A) Confocal images showing the distribution of paxillin (green) and F-actin (red) in sparsely seeded HUVECs. Nuclei were stained with DAPI (blue). Bar, 10 µm. (B) FACS histograms (left) and quantification of mean fluorescence intensity (right) of total integrin β1 (upper panel) and active integrin β1 (lower panel). Quantifications are means + SEM from n=4, normalized against fluorescence intensity of control. (C) Representative Western blot showing β1 expression. Actin was used as a loading control. (D) HUVECs were pretreated with 500 μM Na3VO4 for 30 mins or left untreated, and then received 1 U/ml thrombin for the indicated time-points in the absence or the presence of Na3VO4. Total and (Y783)-phosphorylated integrin β1 were detected by Western blotting, using tubulin as a loading control. *P<0.05 (TIF 966 KB)

Suppl Fig. S3. Arg does not regulate the expression of AJ proteins. (A) Representative Western blots and quantification of VE-cadherin and β-catenin in control versus Arg-depleted HUVECs. Mean ± SEM of n=6 donors. (B) Representative Western blots and quantification of VE-cadherin protein content in lung lysates of WT versus Arg-/- mice. Mean ± SEM of n=6-8 mice per group. NS, not significant (TIF 272 KB)

Suppl Fig. S4. Arg does not regulate leukocyte extravasation during inflammation. (A) Gel electrophoresis of PCR-amplified *Abl2* DNA from wild-type (+/+), heterozygous (+/-) or homozygous (-/-) Arg knock-out mice. (B) Total cell count in broncho-alveolar lavage fluid of mice exposed to intra-tracheal LPS. Lavage was performed 18 hrs after LPS exposure. Mean ± SEM of n=4-6 mice per group. NS, not significant; *P<0.05. (C) Phosphorylation of (Y207)CrkL in control versus Arg-depleted HUVECs under basal conditions and during thrombin (1U/mL) stimulation. Representative blots of n = 3 experiments (TIF 172 KB)

Suppl Fig. S5. Arg is activated in pulmonary endothelium in septic patients. Immunohistochemistry staining of P(Y207)CrkL in paraffin lung slices of all septic versus non-septic patients included in the study. Brown staining indicates a positive staining for P(Y207) CrkL, against a blue background of hematoxillin staining (TIF 1337 KB)

Suppl table S1. Patient characteristics. ARVD = arrhythmogenic right ventricular dysplasia, F = female, M = male. Suppl table S2. Sequences of primers used in this study. *Fw*, forward; *Rev*, reverse. #Sequences kindly provided by C.Britto (Yale University, New Haven), *Sequences kindly provided by A. Koleske (Yale University, New Haven). *TUBB2*, *GAPDH*, and *Rps15* were used as reference genes. Note: The *Adgre1* gene encodes macrophage marker F4/80. Suppl table S3. Sequences of siRNAs and shRNAs used in this study (DOCX 29 KB)

## Data Availability

The datasets generated during and/or analysed during the current study are available from the corresponding author on reasonable request.

## Abbreviations

AJ: Adherens junction
ARDS: Acute respiratory distress syndrome
Arg: Abl-related gene
CrkL: Crk-like
EB: Evans blue
ECIS: Electrical cell-substrate impedance sensing
ECM: Extracellular matrix
FA: Focal adhesion
FB: Fibrillar adhesion
FN: Fibronectin
HSA: Human serum albumin
HPMVEC: Human pulmonary microvascular endothelial cell
HUVEC: Human umbilical vein endothelial cell
LPS: Lipopolysaccharide
MLC: Myosin light chain
pMLC: Phospho-MLC
P(Y): Phosphotyrosine
VE-cadherin: Vascular endothelial cadherin
VEGF: Vascular endothelial growth factor
WT: Wild type
