# Correction to: Depletion of Arg/Abl2 improves endothelial cell adhesion and prevents vascular leak during inflammation

**DOI:** 10.1007/s10456-021-09808-3

**Published:** 2021-08-09

**Authors:** Joana Amado-Azevedo, Anne-Marieke D. van Stalborch, Erik T. Valent, Kalim Nawaz, Jan van Bezu, Etto C. Eringa, Femke P. M. Hoevenaars, Iris M. De Cuyper, Peter L. Hordijk, Victor W. M. van Hinsbergh, Geerten P. van Nieuw Amerongen, Jurjan Aman, Coert Margadant

**Affiliations:** 1 Department of Physiology, Amsterdam Cardiovascular Sciences, Amsterdam University Medical Center, Amsterdam, The Netherlands; 2 Sanquin Research, Amsterdam, The Netherlands; 3 Department of Pulmonology, Amsterdam Cardiovascular Sciences, Amsterdam University Medical Center, Amsterdam, The Netherlands; 4 Angiogenesis Laboratory, Department of Medical Oncology, Cancer Center Amsterdam, Amsterdam University Medical Center, Amsterdam, The Netherlands

## Correction to: Angiogenesis 10.1007/s10456-021-09781-x

In the original article, due to an XML tagging error the equal contribution statement “Joana Amado-Azevedo, Anne-Marieke D. van Stalborch, Erik T. Valent have contributed equally to this work (shared first author), Jurjan Aman and Coert Margadant have contributed equally to this work (shared last author).” was not correctly reflected in the PubMed.

The original article has been corrected.

