# Supplementary figures and images for: Depletion of Arg/Abl2 improves endothelial cell adhesion and prevents vascular leak during inflammation
Source: Angiogenesis. 2021 Mar 26;24(3):677–93. doi: 10.1007/s10456-021-09781-x (PMC7996118; doi:10.1007/s10456-021-09781-x)

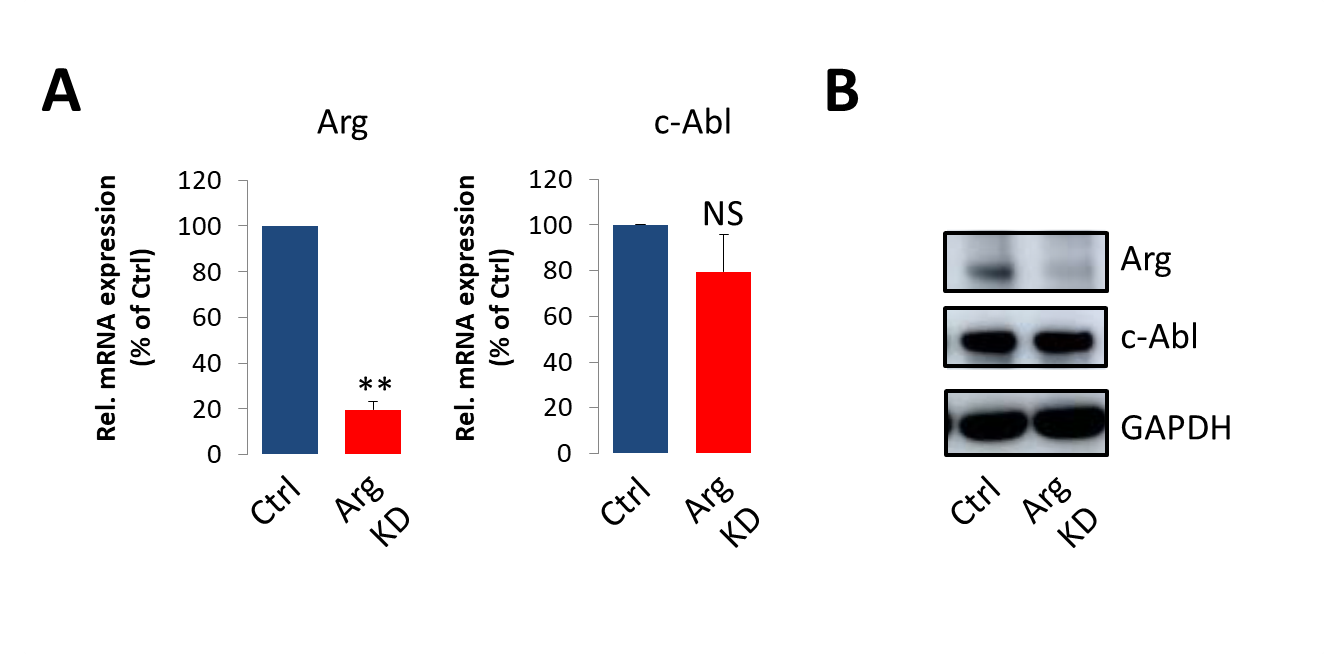

Supplement: Supplementary file 1 — Suppl Fig. S1. Selective knockdown of Arg but not c-Abl in HUVECs. (A) Relative mRNA levels of Arg (left) and c-Abl (right) in HUVECs expressing scrambled sequences (Ctrl) or short hairpins against Arg. Quantifications are means + SEM from n=6, normalized to GAPDH. (B) Western blot showing expression of Arg and c-Abl in HUVECs. GAPDH was used as a loading control. Experiment is representative of n=5 (TIF 89 KB) [file 10456_2021_9781_MOESM1_ESM.tif]

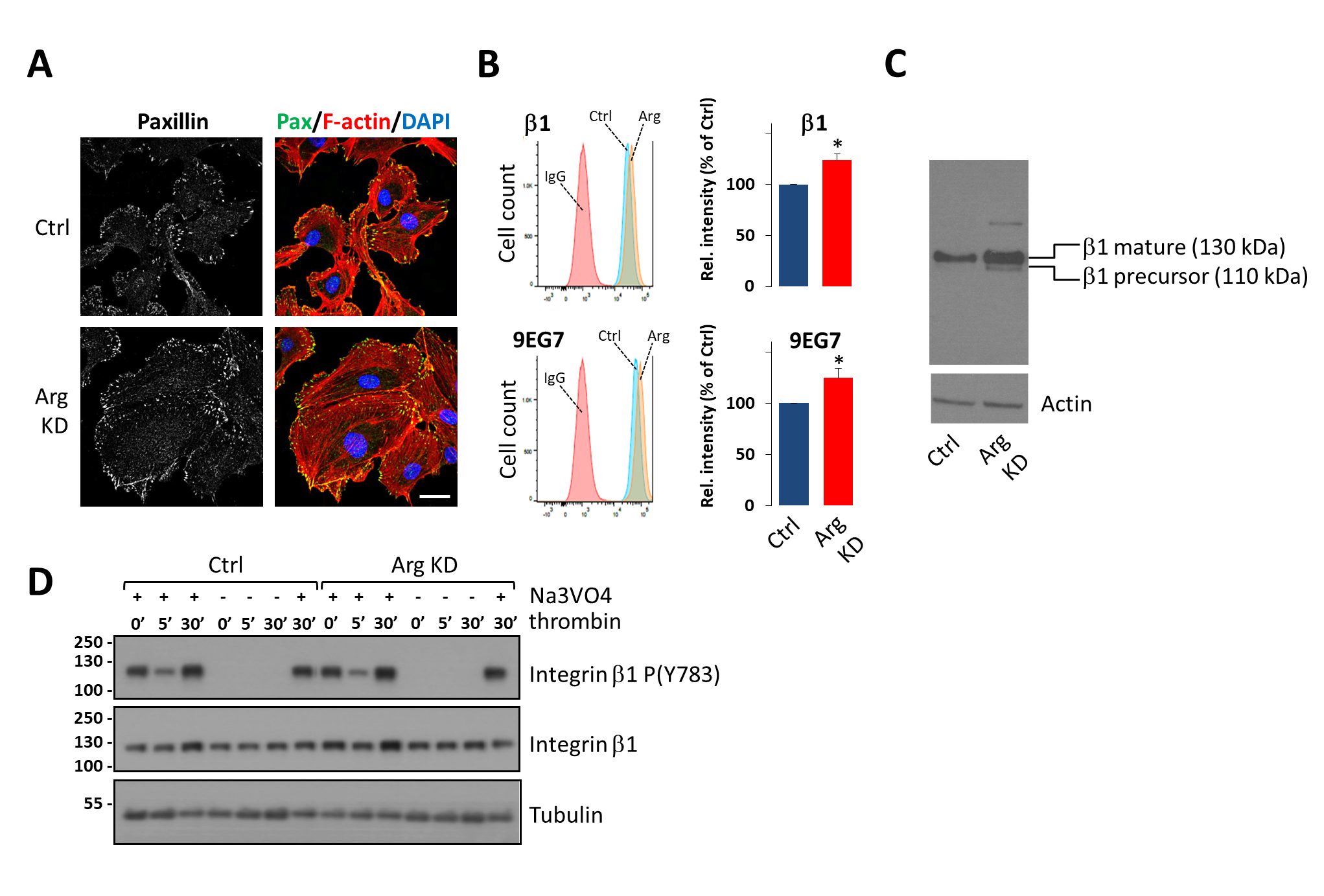

Supplement: Supplementary file 2 — Suppl Fig. S2. Arg controls organization of cell-matrix adhesions, cell spreading, and β1 integrin expression in HUVECs. (A) Confocal images showing the distribution of paxillin (green) and F-actin (red) in sparsely seeded HUVECs. Nuclei were stained with DAPI (blue). Bar, 10 µm. (B) FACS histograms (left) and quantification of mean fluorescence intensity (right) of total integrin β1 (upper panel) and active integrin β1 (lower panel). Quantifications are means + SEM from n=4, normalized against fluorescence intensity of control. (C) Representative Western blot showing β1 expression. Actin was used as a loading control. (D) HUVECs were pretreated with 500 μM Na3VO4 for 30 mins or left untreated, and then received 1 U/ml thrombin for the indicated time-points in the absence or the presence of Na3VO4. Total and (Y783)-phosphorylated integrin β1 were detected by Western blotting, using tubulin as a loading control. *P<0.05 (TIF 966 KB) [file 10456_2021_9781_MOESM2_ESM.tif]

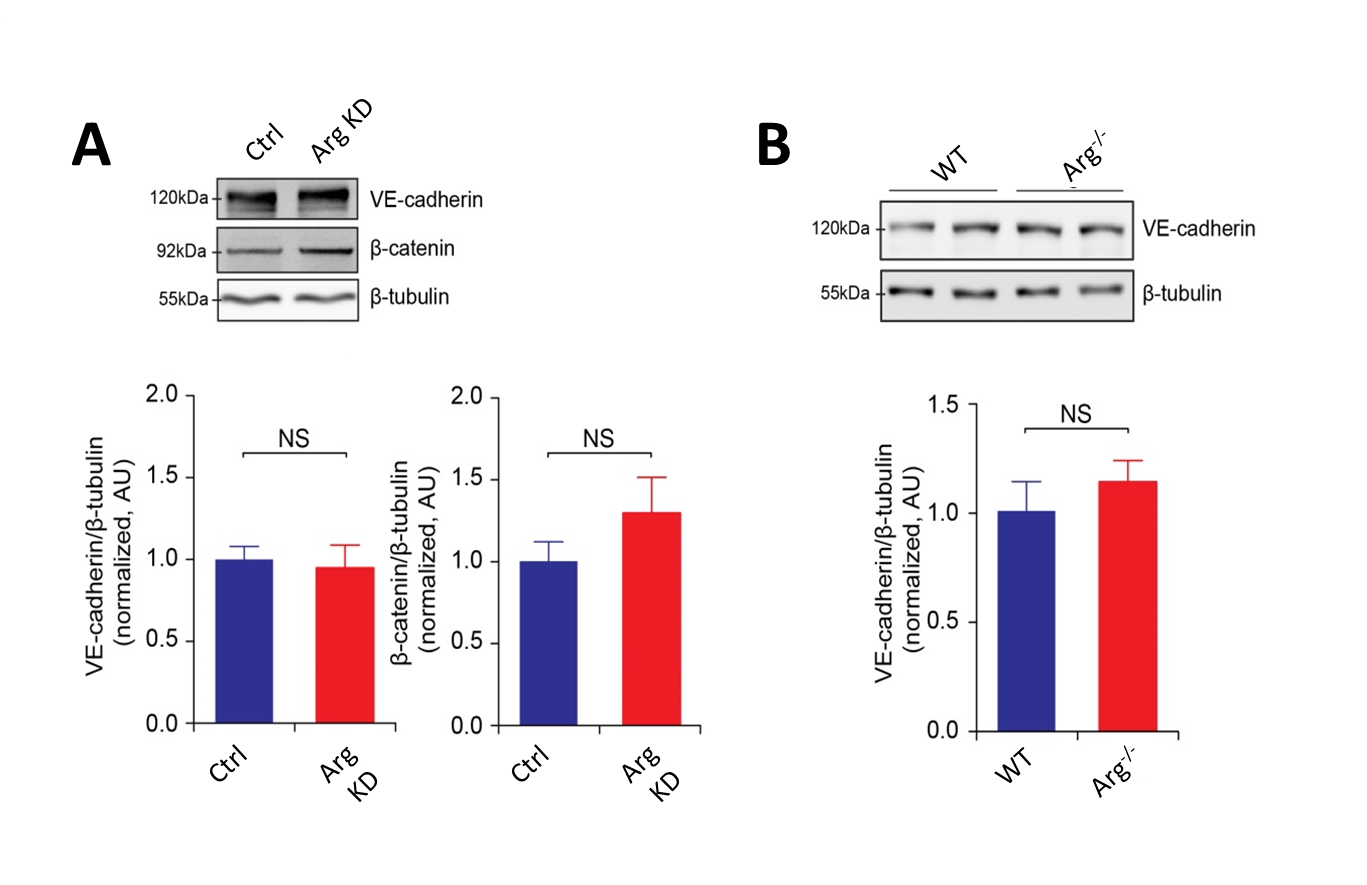

Supplement: Supplementary file 3 — Suppl Fig. S3. Arg does not regulate the expression of AJ proteins. (A) Representative Western blots and quantification of VE-cadherin and β-catenin in control versus Arg-depleted HUVECs. Mean ± SEM of n=6 donors. (B) Representative Western blots and quantification of VE-cadherin protein content in lung lysates of WT versus Arg-/- mice. Mean ± SEM of n=6-8 mice per group. NS, not significant (TIF 272 KB) [file 10456_2021_9781_MOESM3_ESM.tif]

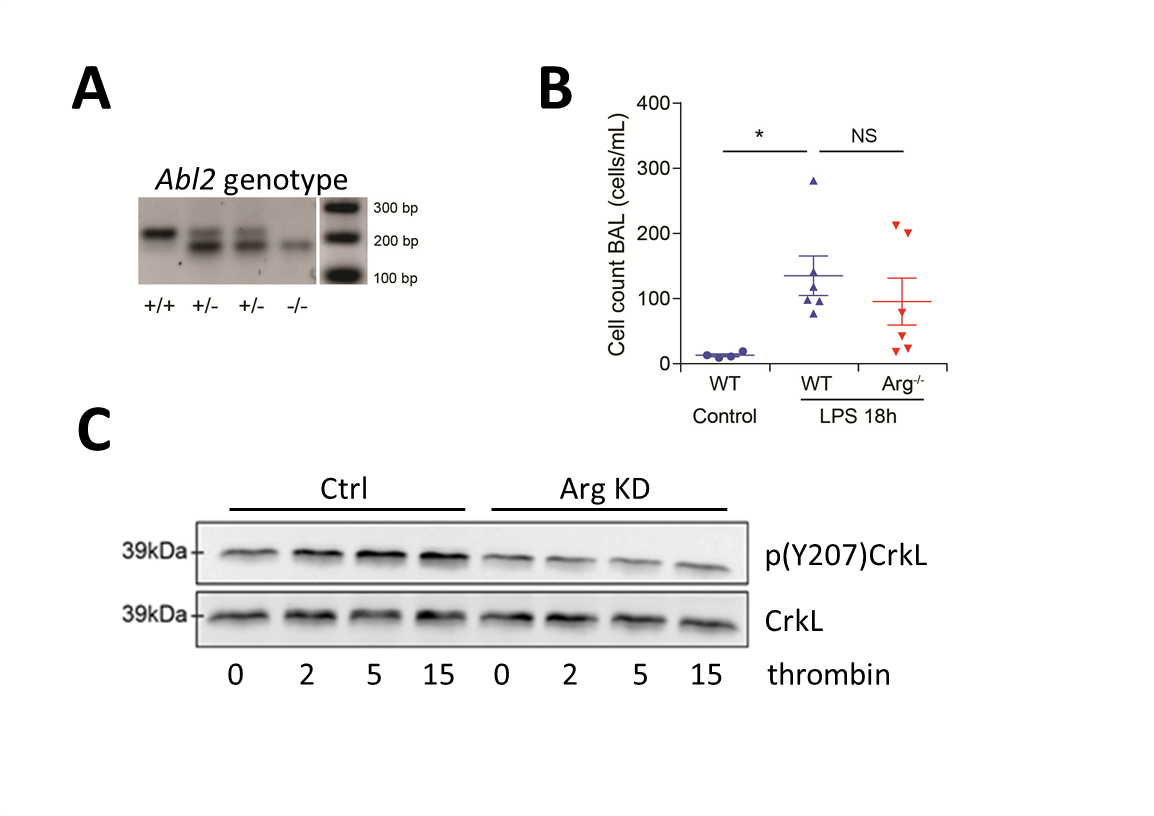

Supplement: Supplementary file 4 — Suppl Fig. S4. Arg does not regulate leukocyte extravasation during inflammation. (A) Gel electrophoresis of PCR-amplified Abl2 DNA from wild-type (+/+), heterozygous (+/-) or homozygous (-/-) Arg knock-out mice. (B) Total cell count in broncho-alveolar lavage fluid of mice exposed to intra-tracheal LPS. Lavage was performed 18 hrs after LPS exposure. Mean ± SEM of n=4-6 mice per group. NS, not significant; *P<0.05. (C) Phosphorylation of (Y207)CrkL in control versus Arg-depleted HUVECs under basal conditions and during thrombin (1U/mL) stimulation. Representative blots of n = 3 experiments (TIF 172 KB) [file 10456_2021_9781_MOESM4_ESM.tif]

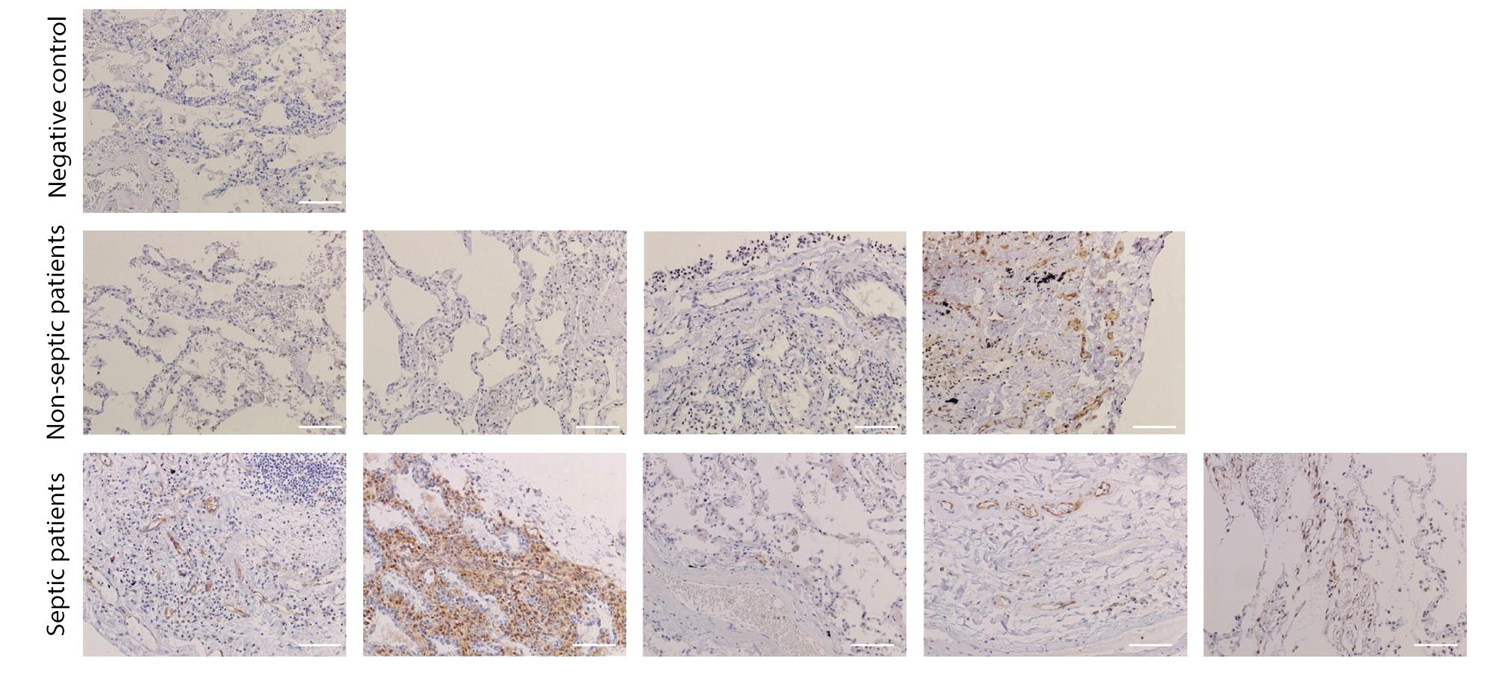

Supplement: Supplementary file 5 — Suppl Fig. S5. Arg is activated in pulmonary endothelium in septic patients. Immunohistochemistry staining of P(Y207)CrkL in paraffin lung slices of all septic versus non-septic patients included in the study. Brown staining indicates a positive staining for P(Y207) CrkL, against a blue background of hematoxillin staining (TIF 1337 KB) [file 10456_2021_9781_MOESM5_ESM.tif]
