# Supplementary material for: Depletion of Arg/Abl2 improves endothelial cell adhesion and prevents vascular leak during inflammation
Source: Angiogenesis. 2021 Mar 26;24(3):677–93. doi: 10.1007/s10456-021-09781-x (PMC7996118; doi:10.1007/s10456-021-09781-x)
Supplement: Supplementary file 6 — Suppl table S1. Patient characteristics. ARVD = arrhythmogenic right ventricular dysplasia, F = female, M = male. Suppl table S2. Sequences of primers used in this study. Fw, forward; Rev, reverse. #Sequences kindly provided by C.Britto (Yale University, New Haven), *Sequences kindly provided by A. Koleske (Yale University, New Haven). TUBB2, GAPDH, and Rps15 were used as reference genes. Note: The Adgre1 gene encodes macrophage marker F4/80. Suppl table S3. Sequences of siRNAs and shRNAs used in this study (DOCX 29 KB) [file 10456_2021_9781_MOESM6_ESM.docx]

**Suppl table S1**

| **Patient** | **Age (yrs)** | **Sex** | **Diagnosis** |
| --- | --- | --- | --- |
| Critically ill, non-septic | 70 | M | Cerebrovascular Accident, ischemic |
|  | 74 | M | Renal failure + chronic heart failure |
|  | 56 | M | Acute myocardial infarction |
|  | 61 | M | Acute myocardial infarction |
|  |  |  |  |
| Critically ill, septic | 76 | M | Pulmonary yeast infection (C.albicans) |
|  | 86 | F | Acute myocardial infarction + Pneumonia |
|  | 66 | M | Bowel necrosis |
|  | 67 | F | ARVD + Pneumonia |
|  |  |  |  |

**Suppl table S2**

| Target | Sequence (5’ to 3’) |
| --- | --- |
| Human |  |
| *ABL1* | *Fw*: CCA GGT GTA TGA GCT GCT AGA G  *Rev*: GTC AGA GGG ATT CCA CTG CCA A |
|  | *Fw*: AAG ACC TTG AAG GAG GAC ACG ATG  *Rev*: CCG TAG GTC ATG AAC TCA GTG ATG |
| *ABL2* | *Fw*: TTC TGG GCA GAG GTA TGG TC  *Rev*: CTG CCT CCA GTC TTG TCT CC |
|  | *Fw*: TCT TCA CCC AGC CTG ATC ACT TTG  *Rev*: GCT CCA CCT GAT AGC CTC ATT TAG |
| *TUBB2* | *Fw*: TTT CAT CCA TCC GAC ATT G  *Rev*: CGG CAG GCA TAC TCA TCT TT |
| *GAPDH* | *Fw*: CTC TCT GCT CCT CCT GTT C  *Rev*: TGA CTC CGA CCT TCA CCT TC |
| Mouse |  |
| *Abl2*^*^ | AAG GGC ATC TCT AAT TGT AAG GAG GAA GG (*primer 1*) |
|  | CTG CAG TGC AAC CCA CGT GTG GGG A (*primer 2*) |
|  | AAT TGA CCT GCA GGG GCC CTC GAC G (*primer 3*) |
| *Adgre1* | *Fw*: CTTTGGCTATGGGCTTCCAGTC  *Rev*: GCAAGGAGGACAGAGTTTATCGTG |
| *Ccl2* | *Fw*: CCACTCACCTGCTGCTACTCAT  *Rev*: TGGTGATCCTCTTGTAGCTCTCC |
| *Il1b* | *Fw*: TGC ACT ACA GGC TCC GAG AT  *Rev*: AGG CCA CAG GTA TTT TGT CGT |
| *Il6*^#^ | *Fw*: CCG GAG AGG AGA CTT CAC AG  *Rev*: TTG CCA TTG CAC AAC TCT TTT |
| *Rps15* | *Fw*: CGG AGA TGG TGG GTA GCA TGG  *Rev*: CGG GTT TGT AGG TGA TGG AGA AC |
| *Tnfa*^#^ | *Fw*: TAG CCC ACG TCG TAG CAA AC  *Rev*: ACA AGG TAC AAC CCA TCG GC |

**Suppl table S3**

| Target | Oligo ID TRCN0000 | Full hairpin sequence |
| --- | --- | --- |
| *ABL2* | 002029 | CCG GCC AGG CAC TAA ATG AGG CTA TCT CGA GAT AGC CTC ATT TAG TGC CTG GTT TTT |
|  | 002030 | CCG GCC TCG TCA TCT GTT GTT CCA TCT CGA GAT GGA ACA ACA GAT GAC GAG GTT TTT |
|  | 002031 | CCG GGC GAA CAG ATA TTA CCA TGA ACT CGA GTT CAT GGT AAT ATC TGT TCG CTT TTT |
|  | 002032 | CCG GCG GTC AGT ATG GAG AGG TTT ACT CGA GTA AAC CTC TCC ATA CTG ACC GTT TTT |
|  | 002033 | CCG GCC CTC AAA CTC GCA ACA AAT TCT CGA GAA TTT GTT GCG AGT TTG AGG GTT TTT |
| *Scrambled* |  | CCG GCA ACA AGA TGA AGA GCA CCA ACT CGA GTT GGT GCT CTT CAT CTT GTT GTT TTT |
| Target |  | **siRNA sequence** |
| *ABL2* | Smartpool | GAA AUG GAG CGA ACA GAU A |
|  | Smartpool | GAG CCA AAU UUC CUA UUA A |
|  | Smartpool | GUA AUA AGC CUA CAG UCU A |
|  | Smartpool | GGA GUG AAG UUC GCU CUA A |
|  | siRNA #1 | GAA AUG GAG CGA ACA GAU A |
|  | siRNA #2 | GAG CCA AAU UUC CUA UUA A |
| *Scrambled* |  | Non-targeting pool #2 from Dharmacon (# D-001206) |
